# Network meta-analysis of triazole, polyene, and echinocandin antifungal agents in invasive fungal infection prophylaxis in patients with hematological malignancies

**DOI:** 10.1186/s12885-021-07973-8

**Published:** 2021-04-14

**Authors:** Huilan Zeng, Zhuman Wu, Bing Yu, Bo Wang, Chengnian Wu, Jie Wu, Jing Lai, Xiaoyan Gao, Jie Chen

**Affiliations:** 1 Department of Hematology, the First Affiliated Hospital of Jinan University, No.613 West Huangpu street, Guangzhou, 510630 P. R. China; 2 Emergency Department, the First Affiliated Hospital of Jinan University, No.613 West Huangpu street, Guangzhou, 510630 P. R. China; 3 Department of Urology Surgery, the First Affiliated Hospital of Jinan University, No.613 West Huangpu street, Guangzhou, 510630 P. R. China

**Keywords:** Echinocandin, Hematological malignancies, Invasive fungal infections, Network meta-analysis, Polyene, Prophylaxis, Triazole

## Abstract

**Background and aim:**

Triazole, polyene, and echinocandin antifungal agents are extensively used to treat invasive fungal infections (IFIs); however, the optimal prophylaxis option is not clear. This study aimed to determine the optimal agent against IFIs for patients with hematological malignancies.

**Methods:**

Randomized controlled trials (RCTs) comparing the effectiveness of triazole, polyene, and echinocandin antifungal agents with each other or placebo for IFIs in patients with hematological malignancies were searched. This Bayesian network meta-analysis was performed for all agents.

**Results:**

The network meta-analyses showed that all triazoles, amphotericin B, and caspofungin, but not micafungin, reduced IFIs. Posaconazole was superior to fluconazole [odds ratio (OR), 0.30; 95% credible interval (CrI), 0.12–0.60], itraconazole (OR, 0.40; 95% CrI, 0.15–0.85), and amphotericin B (OR, 4.97; 95% CrI, 1.73–11.35). It also reduced all-cause mortality compared with fluconazole (OR, 0.35; 95% CrI, 0.08–0.96) and itraconazole (OR, 0.33; 95% CrI, 0.07–0.94), and reduced the risk of adverse events compared with fluconazole (OR, 0.02; 95% CrI, 0.00–0.03), itraconazole (OR, 0.01; 95% CrI, 0.00–0.02), posaconazole (OR, 0.02; 95% CrI, 0.00–0.03), voriconazole (OR, 0.005; 95% CrI, 0.00 to 0.01), amphotericin B (OR, 0.004; 95% CrI, 0.00–0.01), and caspofungin (OR, 0.05; 95% CrI, 0.00–0.42) despite no significant difference in the need for empirical treatment and the proportion of successful treatment.

**Conclusions:**

Posaconazole might be an optimal prophylaxis agent because it reduced IFIs, all-cause mortality, and adverse events, despite no difference in the need for empirical treatment and the proportion of successful treatment.

**Supplementary Information:**

The online version contains supplementary material available at 10.1186/s12885-021-07973-8.

## Background

Adult patients who were diagnosed with hematological malignancies, such as acute lymphoblastic leukemia [1], acute myeloid leukemia [2], or myelodysplastic syndrome, and then instructed to receive intensive chemotherapy for remission or hematopoietic stem cell transplantation (HSCT) were at high risk of developing invasive fungal infections (IFIs) [3, 4], especially *Aspergillus*- and *Candida*-related IFIs [5, 6]. IFIs contribute a lot to the morbidity and mortality in patients with hematological malignancies [4, 7] because the symptoms and signs are absent or nonspecific in the early stage [8, 9]. Thus, antifungal prophylaxis remains central to the containment of IFIs, making the early identification of IFIs difficult [2, 10, 11].

Triazole, polyene, and echinocandin antifungal agents have been extensively applied to prevent and treat IFIs [3]. A large number of clinical trials have been performed to investigate the role of antifungal prophylaxis against IFIs [1, 2, 12–15]. Meanwhile, several meta-analyses have been performed to investigate the comparative efficacy and safety of the treatments [3, 4, 16, 17]. However, the previous meta-analyses were limited by some drawbacks such as insufficient number of eligible studies and treatments. Thus, which treatments should be preferably prescribed to patients who were at high risk of IFIs remained unclear.

The present Bayesian network meta-analysis combined direct and indirect evidence comparing the relative efficacy of all antifungal prophylaxis regimes to determine the optimal agents against IFIs among high-risk patients.

## Methods

This systematic review and network meta-analysis was performed according to the methodology framework recommended by the Cochrane Collaboration, and all summarized results were reported in accordance with the Preferred Reporting Items for Systematic Reviews and Meta-Analyses (PRISMA) statement [18] (Supplementary file 1) and the International Society for Pharmacoeconomics and Outcomes Research Task Force on Indirect Treatment Comparisons Good Research Practices [19]. A formal protocol was not developed for this study.

### Study identification

A systematic search of the databases PubMed, Cochrane Central Register of Controlled Trials, and Embase was conducted to capture all potential studies evaluating the prophylactic use of triazole, polyene, and echinocandin antifungal agents from their inception to April 2020. Each search strategy was modified depending on the specific requirements of the individual database under the assistance of a senior investigator. The reference lists of all eligible studies and topic-related reviews and the clinicaltrials.gov were also searched to include additional studies. The details of all search strategies for the three targeted databases after completing the electronic search are shown in Supplementary file 2. Any disagreement in study identification was resolved by consensus.

### Study selection

Two investigators (Jie Wu and Jing Lai) were assigned to finish the study selection in the following three steps: (a) first, all duplicate records were eliminated using the Duplicates Elimination function of EndNote software; (b) the relevance of each record was evaluated by reviewing title and abstract; and (c) the eligibility of the remaining studies was checked by reviewing the full text eventually. Any divergence in study selection was resolved by consensus. Inclusion and exclusion criteria were developed to guide the study selection. The inclusion criteria were as follows: (a) adult patients with hematological malignancies receiving intensive chemotherapy for remission or HSCT; (b) randomized controlled trials (RCTs) comparing triazole, polyene, and echinocandin antifungal agents with placebo or with each other as prophylaxis against IFIs; (c) the overall incidence of proven or probable IFIs defined as the primary outcome, while the incidence of invasive *Aspergillus* and *Candida* infection, all-cause and IFI-related mortality, overall incidence of adverse events, withdrawal due to adverse events, need for empirical treatments, and proportion of successful treatment regarded as secondary outcomes; and (d) only studies published in English language.

A study was excluded if at least one of the following criteria was met: (a) studies without sufficient data and additional information not added through contacting the lead author and (b) duplicate study with relatively insufficient data.

### Data extraction

Two investigators (Bing Yu and Bo Wang) independently extracted the following information, name of the first author, publication year, study design (multicenter and single center), country of the corresponding author, basic characteristics of participants (sample size, age, and sex ratio), details of treatments, follow-up time, outcomes, and details of the risk of bias. Any divergence in data extraction was resolved by consensus.

### Quality assessment

The quality of eligible studies was assessed with the Cochrane risk-of-bias assessment tool [20] based on the random sequence generation; allocation concealment; blinding of participants and personnel; blinding of outcome assessment; incomplete outcome data; selective reporting; and other bias, which were performed by two independent investigators (Jie Wu and Jing Lai). A study was labeled as a low risk of bias if all items of the assessment tool were covered. A study was rated as a high risk of bias if at least one of the seven items was not fulfilled. Beyond that, a study was labeled as an unclear risk of bias. Any divergence in the quality assessment of studies was settled by consensus.

### Statistical analysis

The data was statistically analyzed by two independent investigators (Zhuman Wu and Chengnian Wu). In this systematic review and network meta-analysis, all outcomes of interest were dichotomous data. Therefore, the pooled risk ratio (RR) with 95% confidence intervals (CIs) was calculated to express it [21]. In a pairwise meta-analysis, heterogeneity across studies was first qualitatively assessed with the Cochrane Q, and then *I*^2^ statistic was used to quantitatively estimate the level of heterogeneity [22]. Studies were deemed to be homogeneous if *P* > 0.1 and *I*^2^ < 50. Otherwise, studies were considered as heterogeneous when *P* < 0.1 and *I*^2^ > 50. All traditional head-to-head meta-analyses were performed with the random-effects model, which simultaneously considered within- and between-study heterogeneity. Publication bias was checked by drawing a funnel plot when the number of eligible studies for individual outcome was more than 10 [23], and an asymmetry suggested publication bias [24]. Traditional pairwise meta-analysis was performed using Review Manager 5.3 (Cochrane Collaboration, Copenhagen, Denmark).

Random-effects network meta-analyses were conducted using Markov Chain Monte–Carlo Methods in OpenBUGS 3.2.3 (MRC Biostatistics Unit, Cambridge, UK) following the methods described by Lu and Ades [25, 26]. The initial value automatically generated from the software was used to fit the model [27]. The Markov Chain Monte–Carlo method with 50,000 iterations and 20,000 burn-in was used to gain convergence. The summary treatment effect estimates were presented as odds ratios (ORs), with 95% credible interval (CrI) for treatment comparisons. The comparison-adjusted funnel plot was drawn to assess the small-study effects when the number of studies included in one pair of comparison was more than 10 [28]. The inconsistency factor was calculated using the loop-specific method to assess the inconsistency [29]. The ranking probabilities of being at each possible rank were estimated for all treatments, and the surface under the cumulative ranking curve values was used to provide a hierarchy of treatments [30].

## Results

### Study selection

**Fig. 1:**
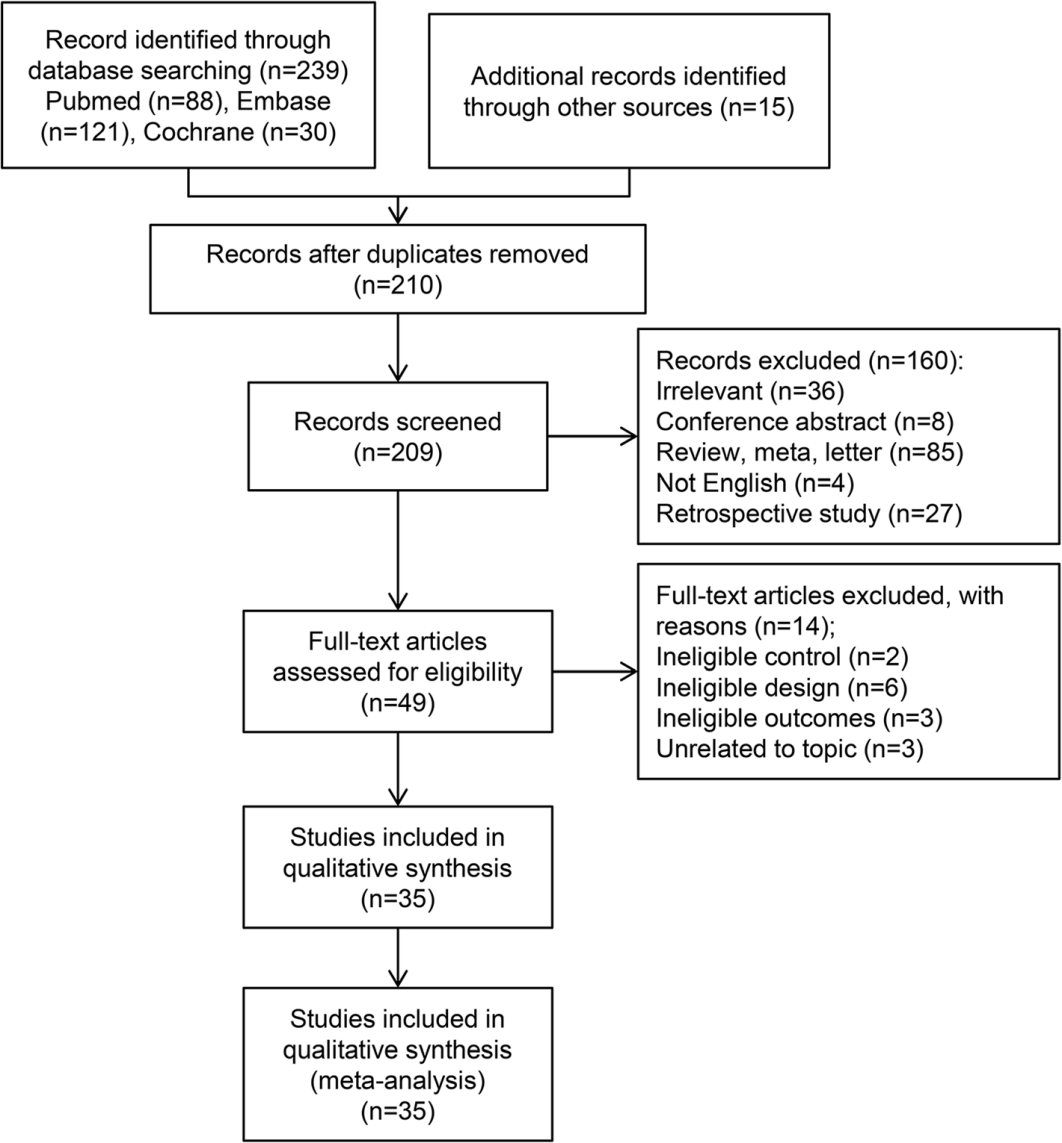
Flow diagram of study retrieval and selection. CENTRAL, Cochrane Central Register of Controlled Trials

The flow diagram of study retrieval and selection is shown in Fig. 1. A total of 239 records were captured after initially searching 3 targeted databases. After removing duplicate records, checking the eligibility of the remaining studies, and then adding additional eligible studies, 35 studies [31–44] involving 37 RCTs were included in this network meta-analysis. The reasons for excluding ineligible studies according to the selection criteria are summarized in Fig. 1.

### Study characteristics

**Fig. 2:**
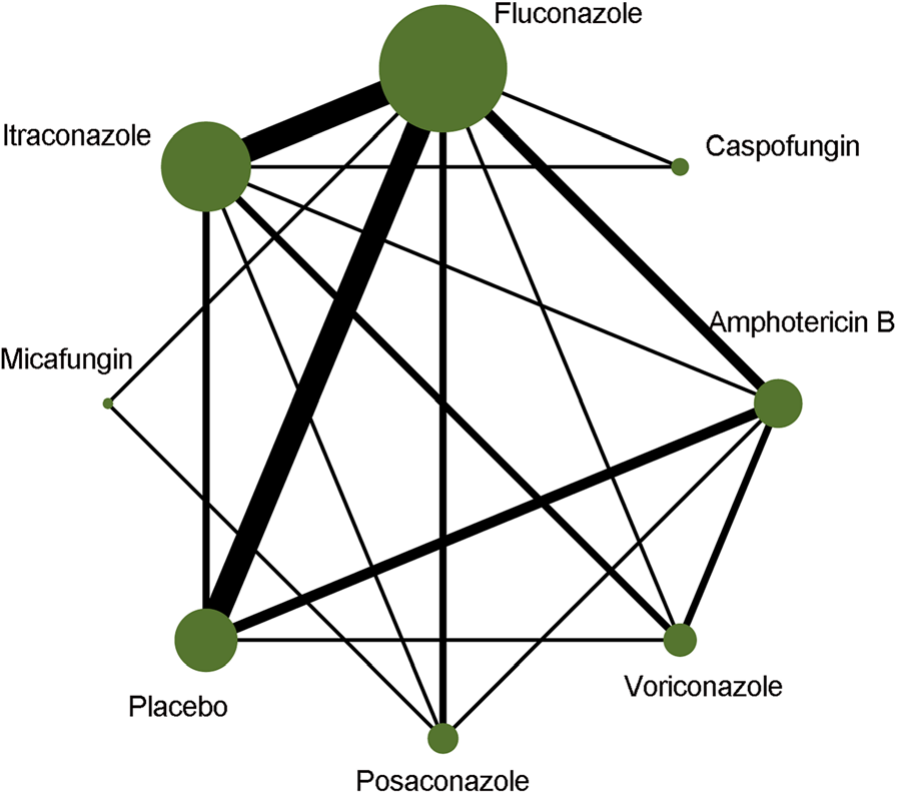
Network of all direct comparisons between antifungal agents and placebo. The sizes of the nodes indicate the numbers of participants, and the widths of the lines indicate the numbers of included trials

The characteristics of all eligible studies are listed in Table 1. Moreover, the details of outcomes of interest are summarized in Table 2. The studies were reported between 1993 and 2019. Of these 35 studies, 16 [1, 2, 38, 40, 43–50] used multiple-center design, 14 [2, 13, 33, 37, 38, 41, 42, 44, 46, 47, 49, 51] did not report details of follow-up, 1 [45] was a three-arm design, and 2 [49, 52] were retrieved from clinicaltrial.gov. The sample size of individual study varied from 25 to 602, with 8513 participants. In total, seven active drugs and placebo were identified. Further, 17 comparisons were identified, and fluconazole was found to be the most extensively studied. The associations among the seven active antifungals and placebo are delineated in Fig. 2.

**Table 1:** Basic characteristics of all eligible studies (*n* = 35)

Study	Country	Design	Sample size	Age (year)	Follow-up	Treatment regimes		
						Group 1	Group 2	Group 3
Cornely 2007	Germany	Multicenter	602 (304 vs 240 vs 58)	(49 ± 17) vs (50 ± 17) vs (52 ± 14)	100 days	Posaconazole 200 mg, oral suspension, thrice daily	Fluconazole 400 mg, oral suspension, once daily	Itraconazole 200 mg, oral solution, twice daily
Cornely 2017	Germany	Multicenter	355 (237 vs 118)	45 (32–57) vs 47 (28–60)	30 days	Amphotericin B 5 mg/kg i.v.	Placebo	n.a.
Epstein 2018	US	Single center	113 (58 vs 55)	61 (32–75) vs 59 (26–74)	12 weeks	Micafungin 100 mg i.v. daily	Posaconazole 400 mg, oral suspension, twice daily	n.a.
Fisher 2019	US	Multicenter	510 (254 vs 256)	10 (0–26) vs 9 (0–21)	n.r.	Caspofungin 70 mg/m^2^ i.v. per day	Fluconazole 6 mg or 12 mg/kg i.v. or oral once daily	n.a.
Mandhaniya 2011	India	Single center	100 (50 vs 50)	5.5 (1.5–15) vs 9 (2–15)	7 days	Voriconazole 6 mg/kg/ to 4 mg/kg twice daily	Amphotericin B 0.5 mg/kg i.v thrice weekly	n.a.
Mattiuzzi 2003	US	Single -center	137 (70 vs 67)	64 (36–83) vs 57 (19–84)	23.3–23.6 months	Amphotericin B 3 mg/kg i.v. per week	Fluconazole capsules 200 mg every 12 h	n.a.
Mattiuzzi 2006	US	Single center	192 (86 vs 106)	60 (17–82) vs 64 (22–82)	n.r.	Itraconazole 200 mg i.v. once daily	Caspofungin 50 mg i.v. once daily	n.a.
Mattiuzzi 2011	US	Single center	123 (71 vs 52)	59 (23–83) vs 60 (21–80)	n.r.	Voriconazole 400 mg every 12 h, followed by 300 mg i.v. twice daily	Itraconazole 200 mg twice daily for 2 days, followed by 200 mg i.v. daily	n.a.
Shen 2013	China	Multicenter	234 (117 vs 117)	40 (17–61) vs 40 (15–68)	100 days	Posaconazole 200 mg, oral suspension, thrice daily	Fluconazole 400 mg once daily	n.a.
Vehreschild 2007	Germany	Multicenter	25 (10 vs 15)	53 (18–73) vs 54 (26–71)	28 days	Voriconazole 200 mg	Placebo	n.a.
Winston 1993	US	Multicenter	256 (124 vs 132)	42.5 (17–82) vs 44.4 (17–73)	90 days	Fluconazole 400 mg once daily	Placebo twice daily	n.a.
Chaftari 2012	US	Single center	40 (19 vs 21)	56 (21–69) vs 55 (20–66)	3 weeks	Amphotericin B 7.5 mg/kg once per week	Posaconazole 200 mg, oral suspension, thrice daily	n.a.
Ellis 1995	Germany	Single center	41 (16 vs 25)	(26.2 ± 13.5) vs (23.6 ± 10.9)	100 days	Fluconazole 8 mg/(kg · day), followed by 4 mg/(kg · day) i.v.	Amphotericin B 1 mg/(kg · day), followed by 0.5 mg/(kg · day)	n.a.
Harousseau 2000	Belgium	Multicenter	557 (281 vs 276)	48 (15–75) vs 49.5 (17–82)	56 days	Itraconazole solution 2.5 mg/(kg · day)	Amphotericin B capsules 500 mg, 4 times/day	n.a.
Laverdiere 2000	Canada	Single center	266 (135 vs 131)	47.6 (18–80) vs 45 (17–77)	n.r.	Fluconazole 400 mg once daily	Placebo once daily	n.a.
Oren 2006	Israel	Single center	195 (99 vs 96)	49 (18–73) vs 50 (17–75)	3 months	Fluconazole 400 mg i.v. or oral daily	Itraconazole 200 mg oral daily, twice daily or 200 mg i.v. daily	n.a.
Rotstein 1999	Canada	Single center	274 (141 vs 133)	47.6 (18–80) vs 45.2 (17–77	n.r.	Fluconazole 400 mg/day	Placebo	n.a.
Slavin 1995	Australia	Single center	300 (152 vs 148)	6.5 (13–60) vs 36.2 (13–65)	110 days	Fluconazole 400 mg/day	Placebo	n.a.
Wingard 2010	US	Multicenter	600 (305 vs 295)	43 (2.7–65.7) vs 43 (9–65)	100 days	Voriconazole 200 mg twice daily	Fluconazole 400 mg per day	n.a.
Annaloro 1995	Italy	Single center	59 (31 vs 28)	30 (17–53) vs 38 (13–56)	n.r.	Itraconazole 400 mg per day	Fluconazole 300 mg per day	n.a.
Chandrasekar 1994	US	Single center	46 (23 vs 23)	39 (17–77) vs 37 (19–67)	70 days	Fluconazole 400 mg per day	Placebo	n.a.
Glasmacher 2006	Germany	Multicenter	494 (248 vs 246)	n.a.	n.r.	Itraconazole oral solution 5 mg/kg	Fluconazole oral solution 400 mg	n.a.
Ito 2007	Japan	Multicenter	209 (103 vs 106)	58 (16–80) vs 53 (16–80)	n.r.	Itraconazole oral capsules 200 mg/day	Fluconazole oral capsules 200 mg/day	n.a.
Marks 2011	UK	Multicenter	489 (224 vs 231)	43.3 (11–70) vs 42.3 (13–70)	28 days	Voriconazole 6 mg i.v., followed by 200 mg	Itraconazole 200 mg, followed by solution 200 mg	n.a.
Marr 2004	US	Single center	299 (148 vs 151)	n.a.	23.3–23.6 months	Fluconazole 400 mg/day, oral or intravenous	Itraconazole oral solution 2.5 mg/kg, thrice daily or intravenous infusion 200 mg per day	n.a.
Menichetti 1999	Italy	Multicenter	405 (201 vs 204)	44 (17–79) vs 44 (17–75)	n.r.	Itraconazole, oral solution, 2.5 mg/kg, 2 times/day	Placebo	n.a.
Nucci 2000	Brazil	Multicenter	210 (104 vs 106)	25.5 (5–63) vs 30 (6–67)	39 days	Itraconazole capsules 100 mg	Placebo	n.a.
Schaffner 1995	Switzerland	Single-center	151 (75 vs 76)	40 (17–71) vs 39 (17–67)	n.r.	Fluconazole oral capsule 400 mg	Placebo	n.a.
Winston 2003	US	Multicenter	38 (71 vs 67)	41 (14–63) vs 38 (17–61)	180 days	Itraconazole 200 mg/12 h for 2 days i.v., followed by i.v. 200 mg/24 h or 200 mg oral solution/12 h	Fluconazole 400 mg intravenously or orally every 24 h	n.a.
Yamac 1995	Turkey	Single center	70 (41 vs 29)	49 (17–68) vs 50 (16–67)	n.r.	Fluconazole oral 400 mg	Placebo	n.a.
Gloria 2012	US	Single center	112 (72 vs 40)	60 (19–84)	42 days	Amphotericin B 3 mg/kg thrice weekly or 9 mg/kg once weekly i.v.	Voriconazole 400 mg, twice daily, followed by 200 mg	n.a.
Mike 2015	UK	Multicenter	355 (237 vs 118)	(44.5 ± 15.16) vs (44.8 ± 17.52)	n.r.	Amphotericin B 5 mg/kg, twice daily	Placebo	n.a.
Karthaus 2000	Germany	Single center	51 (20 vs 31)	42.5 (19–72) vs 46.0 (22–76)	n.r.	Amphotericin B 1 mg/kg i.v. over 4 h every 48 h	Placebo	n.a.
Park 2000	South Korea	Single center	250 (165 vs 85)	46 (20–63) vs 50 (20–64)	100 days	Micafungin 50 mg/day i.v. as a 1-h infusion	Fluconazole oral 400 mg/day	n.a.
Wolff 2000	US	Multicenter	355 (196 vs 159)	43 (20–68) vs 42 (18–59)	n.r.	Fluconazole 400 mg/day oral or i.v.	Amphotericin B 0.2 mg/kg with a maximum dose of 20 mg i.v. per day	n.a.

*n.a.* Not applicable, *n.r.* not reported

**Table 2:** Outcomes of 35 eligible studies

Study	Regimes	Sample size	IFIs			Mortality		AEs		Empirical treatment	Successful treatment
			Probable/Proven	IA	IC	All-cause	IFI-related	All	Withdrawal due to AEs		
Cornely 2007	Posaconazole	304	7	2	3	n.r.	n.r.	159	19	n.r.	n.r.
	Fluconazole	240	19	15	2	n.r.	n.r.	143	4	n.r.	n.r.
	Itraconazole	58	6	5	0	n.r.	n.r.	32	2	n.r.	n.r.
Cornely 2017	Amphotericin B	237	18	n.r.	1	17	2	237	226	37	142
	Placebo	118	13	n.r.	3	8	0	115	110	24	77
Epstein 2018	Micafungin	58	5	2	0	7	2	1	n.r.	n.r.	38
	Posaconazole	55	3	0	1	2	0	17	n.r.	n.r.	26
Fisher 2019	Caspofungin	254	6	2	n.r.	n.r.	n.r.	83	n.r.	160	n.r.
	Fluconazole	256	17	5	n.r.	n.r.	n.r.	98	n.r.	162	n.r.
Mandhaniya 2011	Voriconazole	50	1	n.r.	n.r.	1	n.r.	22	3	11	36
	Amphotericin B	50	0	n.r.	n.r.	2	n.r.	16	15	13	33
Mattiuzzi 2003	Amphotericin B	70	3	n.r.	n.r.	10	1	10	n.r.	n.r.	34
	Fluconazole	67	3	n.r.	n.r.	8	1	5	n.r.	n.r.	32
Mattiuzzi 2006	Itraconazole	86	5	1	4	7	2	n.r.	8	n.r.	44
	Caspofungin	106	7	2	2	7	4	n.r.	4	n.r.	55
Mattiuzzi 2011	Voriconazole	71	0	0	n.r.	6	n.r.	15	n.r.	21	48
	Itraconazole	52	2	1	n.r.	6	n.r.	6	n.r.	20	29
Shen 2013	Posaconazole	117	4	n.r.	n.r.	3	n.r.	25	n.r.	11	80
	Fluconazole	117	11	n.r.	n.r.	7	n.r.	15	n.r.	27	68
Vehreschild 2007	Voriconazole	10	n.r.	n.r.	n.r.	0	n.r.	3	n.r.	n.r.	n.r.
	Placebo	15	n.r.	n.r.	n.r.	2	n.r.	6	n.r.	n.r.	n.r.
Winston 1993	Fluconazole	124	5	3	2	26	n.r.	n.r.	n.r.	n.r.	n.r.
	Placebo	132	10	3	7	24	n.r.	n.r.	n.r.	n.r.	n.r.
Chaftari 2012	Amphotericin B	19	1	n.r.	n.r.	n.r.	n.r.	19	n.r.	n.r.	n.r.
	Posaconazole	21	0	n.r.	n.r.	n.r.	n.r.	20	n.r.	n.r.	n.r.
Ellis 1995	Fluconazole	16	6	4	2	8	5	0	0	n.r.	n.r.
	Amphotericin B	25	3	2	0	6	2	20	3	n.r.	n.r.
Harousseau 2000	Itraconazole	281	8	5	2	18	1	222	13	114	206
	Amphotericin B	276	14	9	3	23	5	205	13	132	198
Laverdiere 2000	Fluconazole	135	9	1	8	n.r.	n.r.	n.r.	n.r.	n.r.	n.r.
	Placebo	131	32	8	23	n.r.	n.r.	n.r.	n.r.	n.r.	n.r.
Oren 2006	Fluconazole	99	12	11	1	11	8	n.r.	n.r.	n.r.	n.r.
	Itraconazole	96	11	9	2	9	3	n.r.	n.r.	n.r.	n.r.
Rotstein 1999	Fluconazole	141	9	n.r.	3	15	1	n.r.	n.r.	n.r.	81
	Placebo	133	32	n.r.	20	15	6	n.r.	n.r.	n.r.	67
Slavin 1995	Fluconazole	152	10	n.r.	n.r.	31	6	n.r.	57	n.r.	n.r.
	Placebo	148	16	n.r.	n.r.	52	13	n.r.	81	n.r.	n.r.
Wingard 2010	Voriconazole	305	22	9	3	n.r.	n.r.	21	n.r.	73	n.r.
	Fluconazole	295	23	17	3	n.r.	n.r.	18	n.r.	89	n.r.
Annaloro 1995	Itraconazole	31	4	n.r.	n.r.	2	n.r.	n.r.	n.r.	16	n.r.
	Fluconazole	28	1	n.r.	n.r.	2	n.r.	n.r.	n.r.	12	n.r.
Chandrasekar 1994	Fluconazole	23	2	2	0	4	2	n.r.	n.r.	n.r.	5
	Placebo	23	1	0	1	2	1	n.r.	n.r.	n.r.	14
Glasmacher 2006	Itraconazole	248	4	2	1	25	2	90	15	n.r.	n.r.
	Fluconazole	246	5	3	1	28	3	61	12	n.r.	n.r.
Ito 2007	Itraconazole	103	1	n.r.	n.r.	n.r.	n.r.	4	n.r.	21	n.r.
	Fluconazole	106	3	n.r.	n.r.	n.r.	n.r.	2	n.r.	20	n.r.
Marks 2011	Voriconazole	224	3	1	2	59	n.r.	n.r.	n.r.	67	109
	Itraconazole	231	5	5	0	80	n.r.	n.r.	n.r.	101	80
Marr 2004	Fluconazole	148	22	7	4	44	11	23	n.r.	25	n.r.
	Itraconazole	151	11	8	3	55	12	52	n.r.	19	n.r.
Menichetti 1999	Itraconazole	201	5	4	1	15	1	n.r.	37	43	166
	Placebo	204	9	1	7	18	5	n.r.	27	59	146
Nucci 2000	Itraconazole	104	5	1	2	8	2	n.r.	6	26	76
	Placebo	106	9	1	6	7	1	n.r.	7	36	63
Schaffner 1995	Fluconazole	75	8	4	4	5	2	n.r.	n.r.	36	n.r.
	Placebo	76	8	7	0	4	2	n.r.	n.r.	25	n.r.
Winston 2003	Itraconazole	71	6	3	2	32	6	33	n.r.	n.r.	n.r.
	Fluconazole	67	17	8	8	28	12	14	n.r.	n.r.	n.r.
Yamac 1995	Fluconazole	41	4	n.r.	n.r.	n.r.	n.r.	n.r.	n.r.	n.r.	n.r.
	Placebo	29	8	n.r.	n.r.	n.r.	n.r.	n.r.	n.r.	n.r.	n.r.
Gloria 2012	Amphotericin B	72	7	n.r.	n.r.	2	n.r.	7	n.r.	n.r.	n.r.
	Voriconazole	40	2	n.r.	n.r.	2	n.r.	4	n.r.	n.r.	n.r.
Mike 2015	Amphotericin B	237	18	n.r.	n.r.	n.r.	n.r.	n.r.	n.r.	n.r.	142
	Placebo	118	12	n.r.	n.r.	n.r.	n.r.	n.r.	n.r.	n.r.	77
Karthaus 2000	Amphotericin B	20	2	n.r.	n.r.	5	0	n.r.	n.r.	9	n.r.
	Placebo	31	6	n.r.	n.r.	9	3	n.r.	n.r.	29	n.r.
Park 2000	Micafungin	165	12	1	1	15	2	n.r.	n.r.	n.r.	155
	Fluconazole	85	7	0	1	11	3	n.r.	n.r.	n.r.	77
Wolff 2000	Fluconazole	196	8	2	8	24	n.r.	n.r.	n.r.	n.r.	103
	Amphotericin B	159	12	1	11	19	n.r.	n.r.	n.r.	n.r.	67

*AEs* Adverse events, *IA* invasive *Aspergillus* infections, *IC* invasive *Candida*, *IFIs* invasive fungal infections, *n.r.* not reported

### Quality assessment

**Fig. 3:**
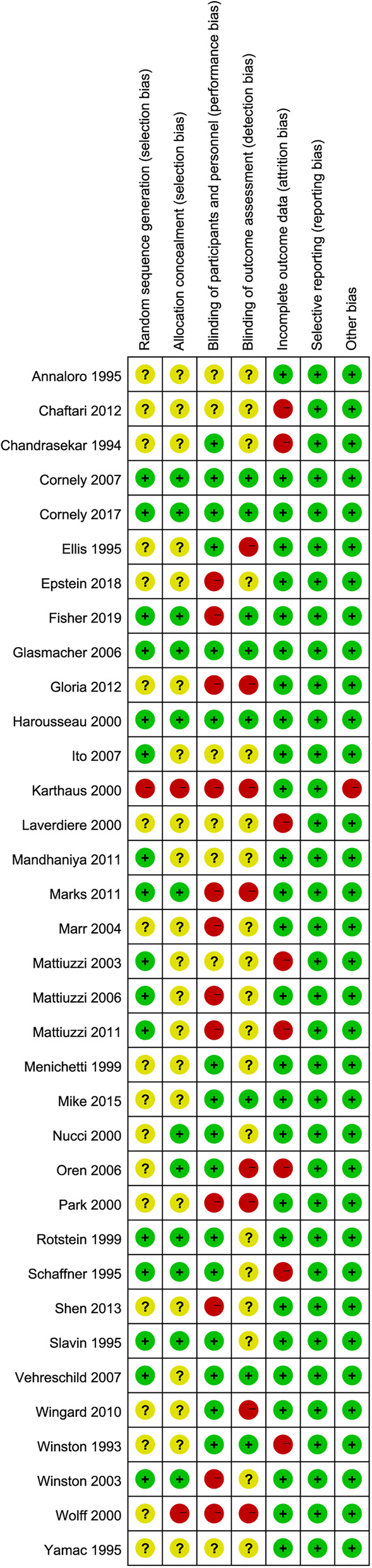
Risk-of-bias summary. The red (−), yellow (?), and green (+) represent high, unclear, and low risk of bias, respectively

The risk of bias of all eligible studies is depicted in Fig. 3. Overall, most of the studies (60.0%) [2, 13, 15, 32, 34, 41, 42, 44, 48, 51–54] had a high risk of bias, and only four studies [1, 40, 45, 46] had a low risk of bias. Of these 35 studies, 19 (54.3%) [12, 14, 15, 31–34, 37, 38, 42–44, 49, 50, 52–55] did not describe the methodology of generating random sequence, 21 (60.0%) [12–15, 31–34, 37, 38, 42, 47, 49–54, 56–58] did not report the details for allocation concealment, and 2 (5.7%) [41, 44] did not conceal random sequence. Four studies (11.4%) [14, 41, 44, 48] used open-label design, and seven (20.0%) [1, 31, 40, 45, 46, 49, 58] reported the details of blinding personnel, participants, and outcome assessors. Attrition bias was detected among eight studies (22.9%) [13, 31, 32, 35, 42, 53, 55, 57] because appropriate methods of addressing incomplete data were not implemented. All studies reported the anticipated outcomes as specified in the Methods section. Other bias sources were not detected in all studies.

### Direct treatment effects

#### Primary outcome

According to the associations among all targeted drugs, direct meta-analyses were performed on proven and probable IFIs, which are delineated in Supplementary file 3 (Fig. S1). Pooled results suggested that fluconazole cloud reduced the incidence of proven and probable IFIs compared with placebo (7 trials; RR, 0.41; 95% CI, 0.25–0.69; *P* < 0.001; *I*^2^ = 37%). However, the effect of fluconazole in reducing proven and probable IFIs was inferior to that of posaconazole (2 trials; RR, 3.17; 95% CI, 1.61–6.23; *P* = 0.008; *I*^2^ = 0%) and caspofungin (1 trial; RR, 2.81; 95% CI, 1.13–7.01; *P* = 0.03; *I*^2^ = n.a.). No significant difference was detected among the remaining comparisons.

#### Secondary outcomes

All comparisons investigating IA-related IFIs are delineated in Supplementary file 3 (Fig. S2). The meta-analysis showed a beneficial result for posaconazole compared with fluconazole (1 trial; RR, 9.50; 95% CI, 2.19–41.14; *P* = 0.003; *I*^2^ = n.a.) and itraconazole (1 trial; RR, 13.57; 95% CI, 2.70–68.23; *P* = 0.002; *I*^2^ = n.a.), but no significant pooled result was detected for other comparisons. Two comparisons reported the incidence of invasive *Candida* (IC)-related IFIs, and meta-analyses suggested that fluconazole (5 trials; RR, 0.34; 95% CI, 0.14–0.85; *P* = 0.02; *I*^2^ = 43%) and itraconazole (2 trials; RR, 0.25; 95% CI, 0.07–0.88; *P* = 0.03; *I*^2^ = 0%) reduced the incidence of IC-related IFIs. All pooled results are displayed in Supplementary file 3 (Fig. S3).

Thirteen comparisons reported the incidence of all-cause mortality, and the meta-analysis did not identify significant differences. All pooled results are delineated in Supplementary file 3 (Fig. S4). Moreover, nine comparisons also reported the incidence of IFI-related mortality. No significant difference was observed among all comparisons, which are delineated in Supplementary file 3 (Fig. S5).

Thirteen comparisons reported the incidence of adverse events. The meta-analysis suggested that fluconazole (5 trials; RR, 0.64; 95% CI, 0.42–0.96; *P* = 0.03; *I*^2^ = 78%) was associated with the reduced incidence of adverse events compared with itraconazole, and micafungin was associated with a reduced incidence of adverse events compared with posaconazole (1 trial; RR, 17.93; 95% CI, 2.47–130.20; *P* = 0.004; *I*^2^ = n.a.). No significant difference was detected among the remaining pooled comparisons. All pooled results are delineated in Supplementary file 3 (Fig. S6). Moreover, 10 comparisons reported withdrawal due to adverse events. The meta-analysis suggested that the incidence of withdrawal due to adverse events in patients receiving fluconazole was lower than that in patients receiving placebo (1 trial; RR, 0.69; 95% CI, 0.53–0.88; *P* = 0.003; *I*^2^ = n.a.) and posaconazole (1 trial; RR, 0.27; 95% CI, 0.09–0.77; *P* = 0.01; *I*^2^ = n.a.). The meta-analysis also indicated a beneficial result for voriconazole (1 trial; RR, 0.20; 95% CI, 0.06–0.65; *P* = 0.007; *I*^2^ = n.a.) compared with amphotericin B for withdrawal due to adverse events. All pooled results are delineated in Supplementary file 3 (Fig. S7).

Ten comparisons reported the need for empirical treatment. Significant differences were detected when fluconazole was related to posaconazole (1 trial; RR, 2.45; 95% CI, 1.28–4.71; *P* = 0.007; *I*^2^ = n.a.), itraconazole was related to voriconazole (2 trials; RR, 1.43; 95% CI, 1.14–1.78; *P* = 0.002; *I*^2^ = 0%) or placebo (2 trials; RR, 0.74; 95% CI, 0.57–0.96; *P* = 0.03; *I*^2^ = 0%), and amphotericin B was related to placebo (2 trials; RR, 0.61; 95% CI, 0.38–0.98; *P* = 0.04; *I*^2^ = 48%). No significant difference was detected among the remaining comparisons. All pooled results are delineated in Supplementary file 3 (Fig. S8).

Eleven comparisons reported the proportion of successful treatment. The meta-analysis suggested that fluconazole was associated with an increased proportion of successful treatment compared with amphotericin B (2 trials; RR, 1.24; 95% CI, 1.02–1.50; *P* = 0.03; *I*^2^ = 0%). Moreover, the meta-analysis also suggested that itraconazole was associated with the increased proportion of successful treatment compared with placebo (2 trials; RR, 1.17; 95% CI, 1.07–1.29; *P* = 0.001; *I*^2^ = 0%); however, itraconazole was inferior to voriconazole (2 trials; RR, 0.75; 95% CI, 0.63–0.90; *P* = 0.002; *I*^2^ = 0%). All pooled results are delineated in Supplementary file 3 (Fig. S9).

### Network meta-analysis

#### Primary outcome

The network meta-analysis was performed to calculate mixed effect estimates. Compared with placebo, fluconazole (OR, 2.19; 95% CrI, 1.39–3.16), itraconazole (OR, 2.92; 95% CrI, 1.64–4.63), posaconazole (OR, 8.51; 95% CrI, 3.25–18.72), voriconazole (OR, 3.40; 95% CrI, 1.41–7.14), amphotericin B (OR, 1.80; 95% CrI, 1.04–2.95), caspofungin (OR, 4.85; 95% CrI, 1.54–11.27), but not micafungin (OR, 3.46; 95% CrI, 0.95–9.06), reduced the incidence of proven and probable IFIs (Table 3). Moreover, the network meta-analysis also suggested that posaconazole was superior to fluconazole (OR, 0.30; 95% CrI, 0.12–0.60), itraconazole (OR, 0.40; 95% CrI, 0.15–0.85), and amphotericin B (OR, 4.97; 95% CrI, 1.73–11.35) in reducing the incidence of proven and probable IFIs.

**Table 3:** Pooled summary estimates derived from direct and network meta-analyses on the comparative efficacy of prophylaxis antifungal agents against IFIs

Comparisons	Direct estimate, OR (95% CI)	Network meta-analysis, OR (95% CrI)
Compared with fluconazole		
Itraconazole	0.71 (0.44–1.15)	0.78 (0.50–1.15)
Posaconazole	**0.32 (0.16–0.62)**	**0.30 (0.12–0.60)**
Voriconazole	0.93 (0.53–1.62)	0.73 (0.31–1.38)
Amphotericin B	0.96 (0.33–2.83)	1.28 (0.71–2.04)
Caspofungin	**0.36 (0.14–0.89)**	0.56 (0.20–1.27)
Micafungin	0.88 (0.36–2.16)	0.84 (0.25–2.11)
Placebo	**2.20 (1.42–3.42)**	**2.19 (1.39–3.16)**
Compared with itraconazole		
Posaconazole	**0.21 (0.08–0.62)**	**0.40 (0.15–0.85)**
Voriconazole	0.48 (0.13–1.72)	0.98 (0.40–1.92)
Amphotericin B	1.78 (0.76–4.18)	1.70 (0.86–2.85)
Caspofungin	1.14 (0.37–3.45)	0.74 (0.26–1.68)
Micafungin	–	1.13 (0.31–2.92)
Placebo	1.77 (0.83–3.76)	**2.92 (1.64–4.63)**
Compared with posaconazole		
Voriconazole	–	2.85 (0.83–7.08)
Amphotericin B	3.30 (0.14–76.46))	**4.97 (1.73–11.35)**
Caspofungin	–	2.20 (0.55–6.24)
Micafungin	1.58 (0.40–6.30)	3.13 (0.85–8.32)
Placebo	–	**8.51 (3.25–18.72)**
Compared with voriconazole		
Amphotericin B	1.40 (0.35–5.52)	1.96 (0.80–4.06)
Caspofungin	–	0.87 (0.23–2.41)
Micafungin	–	1.32 (0.30–4.01)
Placebo	–	**3.40 (1.41–7.14)**
Compared with amphotericin B		
Caspofungin	–	0.47 (0.14–1.20)
Micafungin	–	0.71 (0.19–1.95)
Placebo	1.11 (0.66–1.87)	**1.80 (1.04–2.95)**
Compared with caspofungin		
Micafungin	–	1.88 (0.35–5.81)
Placebo	–	**4.85 (1.54–11.27)**
Compared with micafungin		
Placebo	–	3.46 (0.95–9.06)

Numbers in bold are statistically significant differences

*CI* Confidence interval, *CrI* credible interval, *IFIs* invasive fungal infections, *OR* odds ratio

The hierarchies of all drugs were generated on the basis of SUCRA values for prophylaxis against proven and probable IFIs. The results indicated that posaconazole had the highest probability of being ranked the best (99.2%), followed by voriconazole (77.9%), itraconazole (66.0%), fluconazole (45.1%), caspofungin (44.0%), micafungin (38.5%), and amphotericin B (24.5%). The plot of rankings of all treatments is delineated in Supplementary file 3 (Fig. S10).

#### Secondary outcomes

The network meta-analysis showed that fluconazole (OR, 0.08; 95% CrI, 0.01–0.27), itraconazole (OR, 0.13; 95% CrI, 0.01–0.44), voriconazole (OR, 15.07; 95% CrI, 1.09–76.67), amphotericin B (OR, 38.32; 95% CrI, 2.97–184.9), micafungin (OR, 41.39; 95% CrI, 2.43–212.8), and placebo (OR, 4.78; 95% CrI, 4.08 to 218.0), but not caspofungin (OR, 24.43; 95% CrI, 0.98–139.1), all increased the incidence of IA-related IFIs compared with posaconazole (Table 4). Itraconazole also reduced the incidence of IC-related IFI compared with placebo (OR, 8.27; 95% CrI, 1.51–26.57) (Table 4).

**Table 4:** Pooled relative risk of secondary outcomes based on combined direct and indirect evidence from Bayesian network meta-analysis with different prophylaxis antifungal agents against IFIs among patients at high risk

Comparisons	IA-related IFIs	IC-related IFIs	All-cause mortality	IFI-related mortality	AEs	Withdrawal due to AEs	Empirical treatment	Successful treatment
Compared with fluconazole								
Itraconazole	0.65 (0.37–1.07)	0.55 (0.12–1.47)	1.08 (0.76–1.43)	0.59 (0.23–1.17)	2.37 (0.98–5.08)	2.30 (0.89–5.09)	1.26 (0.49–2.73)	1.41 (0.65–3.13)
Posaconazole	**0.08 (0.01**–**0.27)**	26.57 (0.17–68.36)	**0.35 (0.08**–**0.96)**	1.08 (0.00–5.32)	1.55 (0.34–4.36)	**6.58 (1.07**–**18.26)**	0.55 (0.05–2.07)	1.47 (0.53–3.15)
Voriconazole	0.45 (0.13–1.07)	202.6 (0.18–42.07)	0.78 (0.42–1.32)	n.e.	3.78 (0.96–11.32)	81.54 (0.04–3.19)	0.80 (0.23–1.99)	2.19 (0.83–5.18)
Amphotericin B	1.16 (0.39–2.55)	0.87 (0.08–2.71)	1.05 (0.66–1.53)	0.99 (0.25–2.71)	**4.15 (1.20**–**12.08)**	6.18 (0.91–11.04)	0.96 (0.21–2.54)	0.95 (0.53–1.78)
Caspofungin	0.73 (0.10–2.47)	20.85 (0.02–12.12)	1.03 (0.24–2.83)	2.51 (0.09–12.74)	1.37 (0.11–5.42)	6.30 (0.11–5.02)	1.67 (0.19–5.47)	1.89 (0.40–5.38)
Micafungin	1.27 (0.25–3.94)	1.41 (0.00–8.44)	0.84 (0.34–1.85)	0.67 (0.02–3.04)	**0.12 (0.00**–**0.61)**	n.e.	n.e.	2.53 (0.81, 5.80)
Placebo	1.36 (0.60–2.66)	3.30 (0.77–8.54)	1.17 (0.81–1.6)	1.80 (0.62–3.86)	**0.02 (0.00**–**0.03)**	2.16 (0.79–4.86)	1.85 (0.54–4.77)	1.01 (0.55–2.02)
Compared with itraconazole								
Posaconazole	**0.13 (0.01**–**0.44)**	254.7 (0.34–210.7)	**0.33 (0.07**–**0.94)**	2.44 (0.00–10.53)	0.73 (0.14–2.21)	7.20 (0.48–9.10)	0.56 (0.04–2.22)	1.24 (0.28–3.03)
Voriconazole	0.72 (0.20–1.75)	508.9 (0.38–122.2)	0.73 (0.43–1.17)	n.e.	1.75 (0.42–5.19)	21.0 (0.02–1.40)	0.67 (0.23–1.51)	1.58 (0.77–2.79)
Amphotericin B	1.88 (0.59–4.28)	2.14 (0.18–7.41)	0.99 (0.62–1.49)	1.89 (0.43–5.75)	1.91 (0.53–5.66)	2.10 (0.52–4.22)	0.79 (0.22–1.83)	0.73 (0.37–1.25)
Caspofungin	1.17 (0.16–4.04)	256.1 (0.07–23.31)	0.96 (0.23–2.59)	4.26 (0.19–21.65)	0.70 (0.04–2.90)	1.08 (0.06–1.93)	1.58 (0.13–5.89)	1.20 (0.37–2.94)
Micafungin	2.12 (0.36–6.92)	6.64 (0.01–22.84)	0.80 (0.29–1.86)	1.43 (0.04–6.53)	**0.06 (0.00**–**0.29)**	n.e.	n.e.	2.12 (0.43–5.55)
Placebo	2.18 (0.94–4.36)	**8.27 (1.51**–**26.57)**	1.11 (0.73–1.59)	**3.39 (1.07**–**8.30)**	**0.01 (0.00**–**0.02)**	1.01 (0.42–2.02)	1.54 (0.55–3.52)	0.77 (0.42–1.34)
Compared with posaconazole								
Voriconazole	**15.07 (1.09**–**76.67)**	189.5 (0.02–46.78)	3.34 (0.67–10.58)	n.e.	3.77 (0.49–14.80)	625.1 (0.01–1.08)	4.33 (0.24–16.63)	1.88 (0.44–5.71)
Amphotericin B	**38.32 (2.97**–**184.9)**	0.94 (0.00–5.56)	**4.49 (1.04**–**13.86)**	136.8 (0.13–579.6)	4.38 (0.61–16.00)	19.4 (0.12–4.10)	12.71 (0.24–19.62)	0.81 (0.25–2.23)
Caspofungin	24.43 (0.98–139.1)	6.18 (0.00–11.56)	4.34 (0.54–16.84)	504.4 (0.10–1102.0)	1.59 (0.06–6.46)	2.66 (0.02–1.59)	86.88 (0.25–37.39)	1.73 (0.23–5.55)
Micafungin	**41.39 (2.43**–**212.8)**	0.73 (0.00–4.27)	3.23 (0.85–9.95)	18.94 (0.13–90.85)	**0.07 (0.00**– –**0.37)**	n.e.	n.e.	1.89 (0.65–4.35)
Placebo	**44.78 (4.08**–**218.0)**	3.67 (0.03–19.83)	**4.98 (1.16**–**15.28)**	247.5 (0.7–1074.0)	**0.02 (0.00**–**0.03)**	0.95 (0.09–2.10)	10.07 (0.59–39.75)	0.86 (0.27–2.47)
Compared with voriconazole								
Amphotericin B	3.42 (0.66–10.49)	1.30 (0.01–4.60)	1.45 (0.71–2.56)	n.e.	1.35 (0.36–3.87)	**14.84 (1.40**–**48.77)**	1.38 (0.33–3.58)	0.50 (0.22–1.01)
Caspofungin	2.19 (0.20–9.42)	5.68 (0.00–11.71)	1.41 (0.31–3.99)	n.e.	0.53 (0.02–2.33)	72.68 (0.13–27.56)	3.44 (0.20–11.09)	0.85 (0.21–2.45)
Micafungin	3.95 (0.47–14.71)	1.89 (0.00–7.79)	1.18 (0.37–2.91)	n.e.	**0.05 (0.00**–**0.23)**	n.e.	n.e.	1.47 (0.27–4.18)
Placebo	3.99 (0.97–1.77)	3.81 (0.05–17.34)	1.62 (0.82–2.84)	n.e.	**0.005 (0.00**–**0.01)**	18.98 (0.65–40.86)	2.81 (0.72–7.96)	0.54 (0.23–1.14)
Compared with amphotericin B								
Caspofungin	0.79 (0.08–3.19)	60.34 (0.04–32.83)	1.02 (0.23–3.09)	4.69 (0.09–18.36)	0.51 (0.02–1.91)	0.92 (0.03–1.78)	2.89 (0.17–11.45)	1.89 (0.46–4.99)
Micafungin	1.41 (0.19–5.25)	6.90 (0.01–21.80)	0.84 (0.30–1.96)	1.01 (0.02–5.01)	**0.04 (0.00**–**0.19)**	n.e.	n.e.	2.95 (0.71–7.23)
Placebo	1.45 (0.39–4.01)	8.07 (0.83–33.43)	1.15 (0.71–1.77)	2.47 (0.53–7.03)	**0.004 (0.00**–**0.01)**	0.79 (0.22–1.79)	2.36 (0.76–6.55)	1.10 (0.65–1.83)
Compared with caspofungin								
Micafungin	3.43 (0.23–15.46)	38.0 (0.00–45.54)	1.21 (0.20–4.19)	1.78 (0.01–8.50)	0.66 (0.00–1.35)	n.e.	n.e.	2.43 (0.28–7.80)
Placebo	3.56 (0.44–13.41)	185.0 (0.19–117.2)	1.66 (0.39–4.83)	3.78 (0.11–18.72)	**0.05 (0.00**–**0.42)**	38.59 (0.40–17.52)	2.96 (0.22–11.86)	0.87 (0.22–2.37)
Compared with micafungin								
Placebo	1.79 (0.27–5.89)	435.6 (0.27–743.0)	1.67 (0.59–3.60)	15.13 (0.42–75.43)	1.39 (0.00–1.52)	n.e.	n.e.	0.53 (0.15–1.60)

The column treatment is compared with the row treatment (i.e., row treatment is the reference for each comparison). Numbers in parentheses indicate 95% credible interval. Numbers in bold represent statistically significant results

*AEs* Adverse events, *IA* invasive *Aspergillus* infections, *IC* invasive *Candida*, *IFIs* invasive fungal infections, *n.e.* not estimated

The network meta-analysis demonstrated that fluconazole (OR, 0.35; 95% CrI, 0.08–0.96), itraconazole (OR, 0.33; 95% CrI, 0.07–0.94), amphotericin B (OR, 4.49; 95% CrI, 1.04–13.86), and placebo (OR, 4.98; 95% CrI, 1.16–15.28), but not voriconazole (OR, 3.34; 95% CrI, 0.67–10.58), caspofungin (OR, 4.34; 95% CrI, 0.54–16.84), and micafungin (OR, 3.23; 95% CrI, 0.85–9.95), increased all-cause mortality compared with posaconazole (Table 4). Itraconazole also reduced the incidence of IFI-related mortality compared with placebo (OR, 3.39; 95% CrI, 1.07–8.30) (Table 4).

The network meta-analysis showed that fluconazole (OR, 0.02; 95% CrI, 0.00–0.03), itraconazole (OR, 0.01; 95% CrI, 0.00–0.02), posaconazole (OR, 0.02; 95% CrI, 0.00–0.03), voriconazole (OR, 0.005; 95% CrI, 0.00–0.01), amphotericin B (OR, 0.004; 95% CrI, 0.00–0.01), and caspofungin (OR, 0.05; 95% CrI, 0.00–0.42), but not micafungin (OR, 1.39; 95% CrI, 0.00–1.52), were associated with a reduced incidence of adverse events compared with placebo (Table 4). Fluconazole (OR, 0.12; 95% CrI, 0.00–0.61), itraconazole (OR, 0.06; 95% CrI, 0.00–0.29), posaconazole (OR, 0.07; 95% CrI, 0.00–0.37), voriconazole (OR, 0.05; 95% CrI, 0.00–0.23), and amphotericin B (OR, 0.04; 95% CrI, 0.00–0.19), but not caspofungin (OR, 0.66; 95% CrI, 0.00–1.35), reduced the incidence of adverse events compared with micafungin. Moreover, fluconazole was associated with an increased incidence of adverse events compared with amphotericin B (OR, 4.15; 95% CrI, 1.20–12.08). The network meta-analysis also demonstrated that fluconazole and voriconazole were superior to posaconazole (OR, 6.58; 95% CrI, 1.07–18.26) and amphotericin B (OR, 14.84; 95% CrI, 1.40–48.77), respectively (Table 4).

The network meta-analysis showed no significant difference among all comparisons in terms of the need for empirical treatment and the proportion of successful treatment (Table 4).

### Publication bias and network coherence

**Fig. 4:**
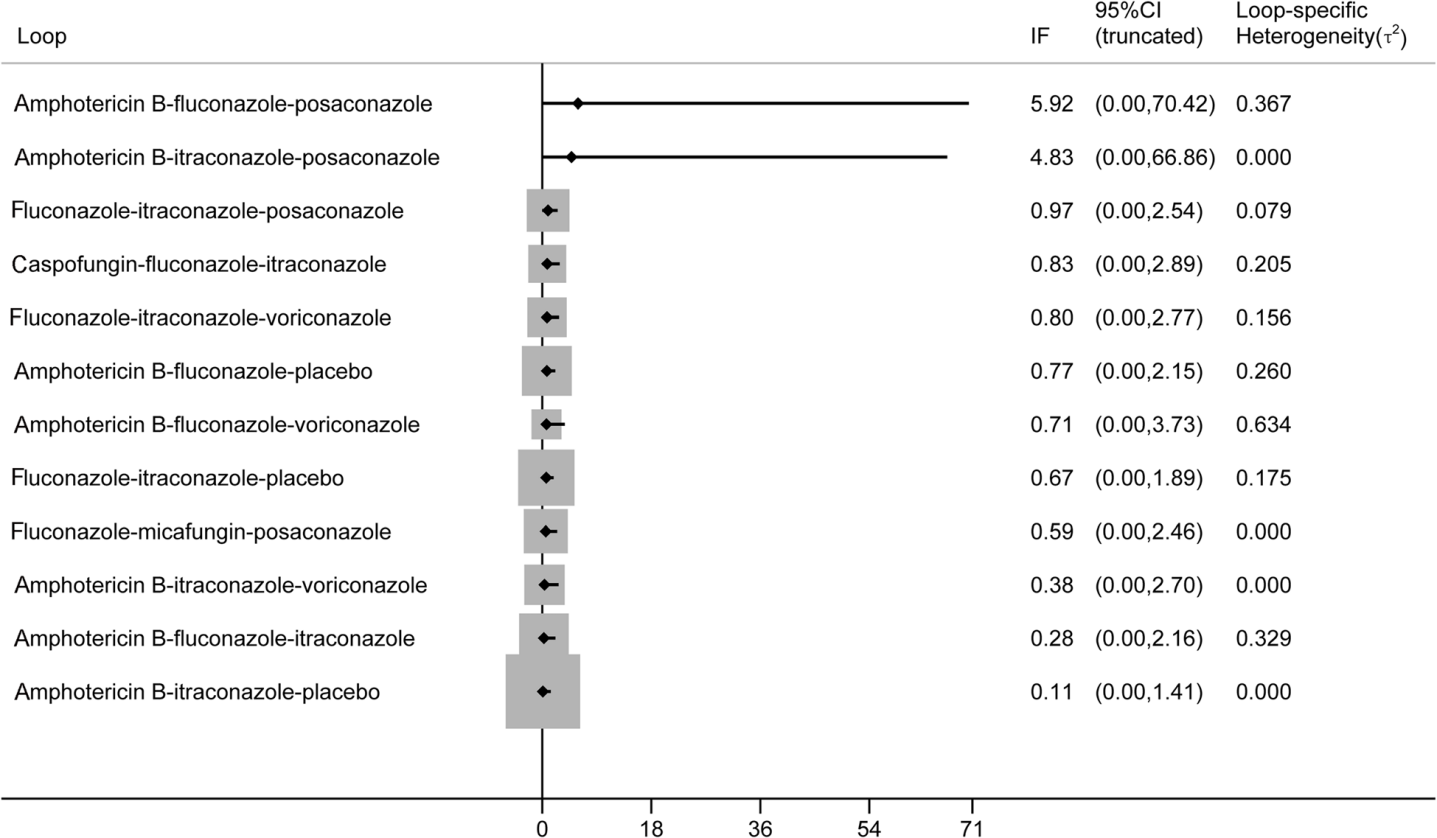
Inconsistency plot of proven and probable IFIs. The lower boundary of confidence interval, including zero, indicates the absence of inconsistency. CI, Confidence interval; IFIs, invasive fungal infections

**Fig. 5:**
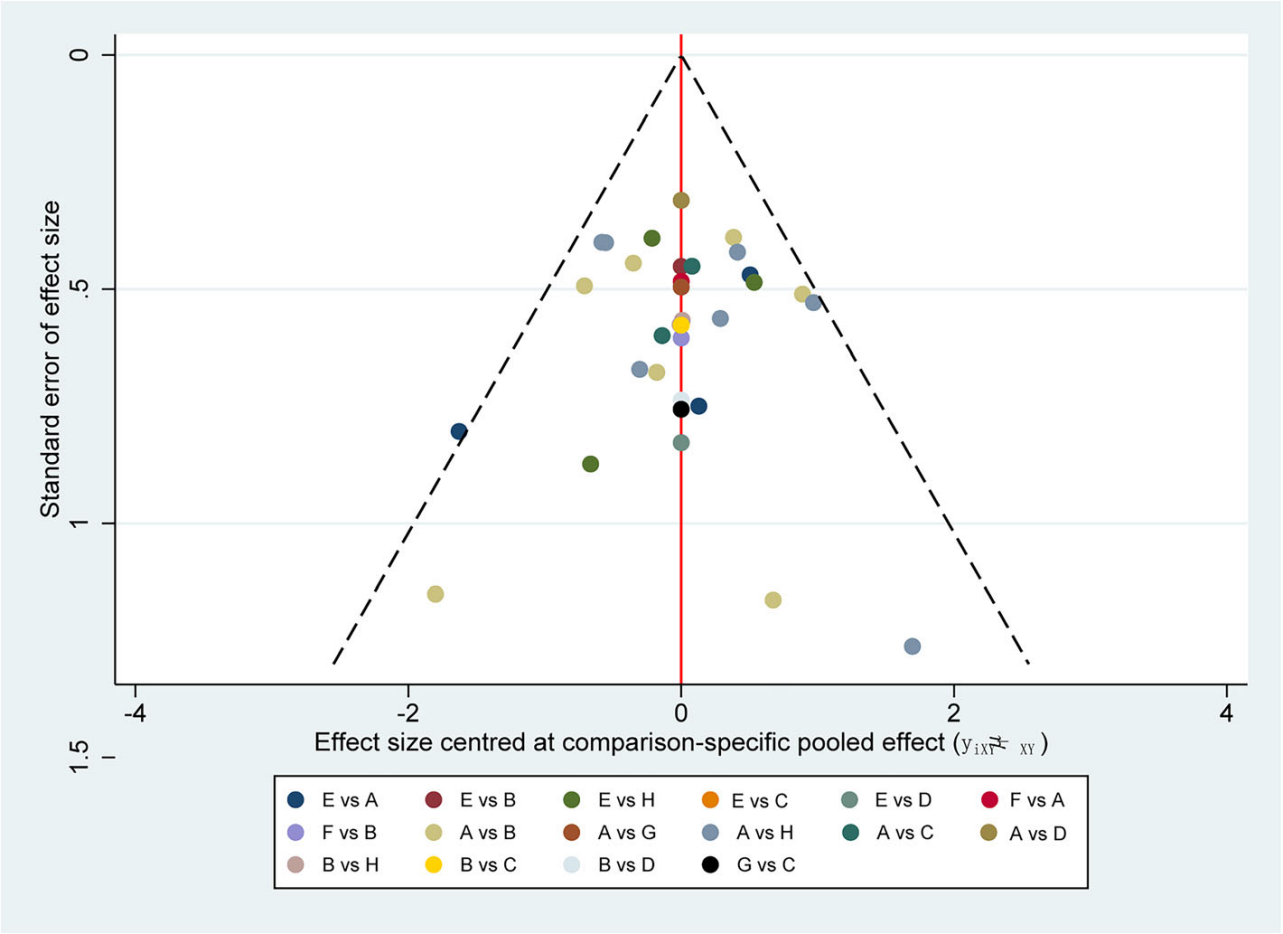
Comparison-adjusted funnel plot for proven and probable IFIs. The vertical axis represents the standard error of effect size, and *x* axis indicates effect size centered at comparison-specific pooled effect. Symmetrical funnel plot indicates the absence of publication bias. IFIs, Invasive fungal infections; A, fluconazole; B, itraconazole; C, posaconazole; D, voriconazole; E, amphotericin B; F, caspofungin; G, micafungin; and H, placebo

The split-node method was adopted to generate the inconsistency plot so as to check the consistency of results from direct and indirect comparisons. The results of inconsistency plot indicated consistency in terms of proven and probable IFIs (Fig. 4). No evidence of publication bias based on comparison-adjusted funnel plot asymmetry was found (Fig. 5), although the number of studies included in each comparison was very small, thereby making the available methods for evaluating publication bias somewhat unreliable.

## Discussion

IFIs remain a leading cause of morbidity and mortality among patients at high risk [4, 7] due to elusive identification of IFIs in the early stage [8, 9]. Therefore, prophylaxis strategies are crucial in the containment of IFIs [3]. Previous traditional direct meta-analyses and network meta-analyses did not consider all prophylaxis treatments and did not incorporate all potentially eligible studies, thus restricting the reference value of previous findings for making decisions in clinical practice. The present network meta-analysis was performed on 35 studies, including 37 RCTs involving 8513 patients, to generate more comprehensive and reliable results.

The valuable findings of this network meta-analysis were as follows: (a) fluconazole, itraconazole, posaconazole, voriconazole, amphotericin B, and caspofungin, but not micafungin, had the potential of reducing the incidence of proven and probable IFIs; (b) posaconazole was superior to fluconazole, itraconazole, and amphotericin B against proven and probable IFIs; (c) posaconazole was superior to fluconazole, itraconazole, voriconazole, amphotericin B, micafungin, and placebo against IA-related IFIs, and itraconazole had the potential of reducing IC-related IFIs; (d) posaconazole was superior to fluconazole, itraconazole, and amphotericin B in terms of all-cause mortality; (e) fluconazole, itraconazole, posaconazole, voriconazole, amphotericin B, or caspofungin had the potential of reducing the risk of adverse events; (f) fluconazole, itraconazole, posaconazole, voriconazole, and amphotericin B were superior to micafungin in reducing the risk of adverse events, and fluconazole and voriconazole were superior to posaconazole and amphotericin B; (g) all treatments were not different in terms of the need for empirical treatment and the proportion of successful treatment; and (h) posaconazole had the highest probability of being ranked the best against proven and probable IFIs.

To date, four topic-related meta-analyses [3, 4, 16, 17] included two traditional pairwise meta-analyses [16, 17] and two network meta-analyses [3, 4]. In 2002, Bow and colleagues [16] performed a meta-analysis of randomized controlled clinical trials to investigate the overall clinical efficacy of antifungal prophylaxis, including azole antifungal agents and low-dose intravenous amphotericin B, for severely neutropenic chemotherapy recipients. The aforementioned analysis included 38 eligible studies and showed that antifungal prophylaxis could reduce all-cause mortality and IFI-related mortality. However, the efficacy and safety of individual antifungal prophylaxis agents were not investigated, thereby mitigating the reference value of the findings. On the contrary, the present analysis explored pure efficacy and safety of individual agents against IFIs and suggested that posaconazole was associated with the reduced incidence of all-cause mortality. In 2005, Vardakas and colleagues [17] separately investigated the comparative efficacy of fluconazole versus itraconazole for antifungal prophylaxis in neutropenic patients with hematological malignancies. The pooled results based on five RCTs suggested that itraconazole was more effective than fluconazole in preventing IFIs in neutropenic patients with hematological malignancies; however, it was also associated with more adverse effects. The present analysis incorporated 35 studies involving 37 RCTs to estimate the mixed efficacy of antifungal prophylaxis agents and found no significant difference between fluconazole and itraconazole in terms of the incidence of IFIs, mortality, and adverse events; the need for empirical treatment; and the proportion of successful treatment. In 2011, Freemantle et al. [4] compared between a systematic review and mixed treatment to investigate the potential of empirical, pre-emptive, and directed treatment strategies for invasive mold infections. This study suggested that caspofungin was superior to amphotericin B and voriconazole in the outcome of survival, and voriconazole was superior to amphotericin B for overall survival. However, the present study found no difference among caspofungin, amphotericin B, and voriconazole in terms of mortality. In 2016, Zhao and colleagues published a network meta-analysis [3] and found that all triazole antifungals were effective in preventing IFIs, which was consistent with the findings of the present analysis. Better than Zhao’s network meta-analysis, the present analysis also suggested that amphotericin B and caspofungin were effective against IFIs. Moreover, Zhao et al. found that posaconazole was more efficacious in reducing IFIs and all-cause death compared with fluconazole and itraconazole, which were also consistent with the findings of the present analysis.

The strength of this meta-analysis included the comprehensive and simultaneous assessment of the relative efficacy of all treatments against IFIs among patients at high risk. Given limited comparative effectiveness studies, it was difficult for patients and physicians to make informed decisions regarding which treatments were the most effective against IFIs. However, the meta-analysis had certain limitations related to both network analysis and individual studies, which merits further discussion. First, direct comparative effectiveness studies were scarce. Second, network meta-analyses might be susceptible to misinterpretation. The biggest threat to the validity of a network meta-analysis was conceptual heterogeneity involving considerable differences in participants, interventions, and specified regimes of targeted treatments, thus limiting the comparability of trials. It was assumed that patients enrolled in all included studies were sampled from the same theoretical population [59, 60]. However, subtle differences were found in characteristics related to patients (adult patients, pediatric patients, patients receiving intensive chemotherapy for remission, and patients undergoing HCST), treatments (dose or form of individual treatment), and administration of agents (intravenous and oral). Third, ranking probabilities might be challenging to understand and did not always imply a clinically important difference. Hence, clinical decisions based on the findings should be made cautiously.

The individual studies included in the analysis also had some limitations, which also undermined the strength of the meta-analysis. Most of the studies focused on the efficacy against IFIs, with very few studies on mortality and adverse events, which limited the assessment of benefits of treatments, and hence a thorough assessment of risk–benefit profile could not be performed. Studies were also under the risk of detection bias with the suboptimal reporting of blinding of outcome assessors. Various study designs, including multicenter and single center, were used in different eligible studies. However, further sensitivity analysis or subgroup analysis was not designed based on the study design due to an insufficient number of eligible studies for the majority of comparisons. Therefore, it was critical to further investigate the impact of study design on pooled results when a sufficient number of eligible studies were published. Moreover, subgroup or sensitivity analysis was not designed according to the follow-up time due to an insufficient number of eligible studies for individual comparison. However, the time effects of treatments were investigated in individual studies, and no novel findings were reported [13, 51].

## Conclusions

Despite these limitations, the present network meta-analysis provided a better understanding of the comparative efficacy of all potential treatments against IFIs among patients who were at high risk. Posaconazole might be a promising option against IFIs because it was superior to fluconazole, itraconazole, amphotericin B, voriconazole, or micafungin, although no significant difference was detected compared with caspofungin in terms of proven and probable IFIs and IA-related IFIs. Moreover, posaconazole also reduced all-cause mortality compared with fluconazole and itraconazole, and reduced the risk of adverse events compared with amphotericin B, fluconazole, itraconazole, posaconazole, voriconazole, amphotericin B, and caspofungin, although all treatments showed no significant difference in terms of the need for empirical treatment and the proportion of successful treatment.

## Supplementary Information

**Additional file 1.** PRISMA 2009 checklist

**Additional file 2.** Details of all targeted databases

**Additional file 3: Fig. S1.** Forest plot of possible and proven IFI**. Fig. S2**. Forest plot of IA-related possible and proven IFI. **Fig. S3**. Forest plot of IC-related possible and proven IFI. **Fig. S4**. Forest plot of all cause mortality. **Fig. S5**. Forest plot of IFI-related mortality. **Fig. S6**. Forest plot of AE. **Fig. S7**. Forest plot of withdrawal due AE. **Fig. S8**. Forest plot of empirical treatment. **Fig. S9**. Forest plot of successful treatment. **Fig. S10**. SUCRA of all drugs for proven and probable IFI

## Data Availability

All data generated or analyzed during this study are included in this published article [and its supplementary information files].

## Abbreviations

IFIs: Invasive fungal infections
OR: Odds ratio
CrI: Credible interval
HSCT: Hematopoietic stem cell transplantation
RCTs: Randomized controlled trials
CIs: Confidence intervals
RR: Risk ratio
