# Supplementary material for: Network meta-analysis of triazole, polyene, and echinocandin antifungal agents in invasive fungal infection prophylaxis in patients with hematological malignancies
Source: BMC Cancer. 2021 Apr 14;21:404. doi: 10.1186/s12885-021-07973-8 (PMC8048157; doi:10.1186/s12885-021-07973-8)
Supplement: Supplementary file 2 — Additional file 2. Details of all targeted databases [file 12885_2021_7973_MOESM2_ESM.docx]

1. **PubMed search strategy**

| Search number | Query |
| --- | --- |
| 18 | (((("Pre-Exposure Prophylaxis"[Mesh]) OR ((((Pre Exposure Prophylaxis[Title/Abstract]) OR (Pre-Exposure Prophylaxi[Title/Abstract])) OR (Pre-Exposure Prophylaxis[Title/Abstract])) OR (Pre Exposure Prophylaxis[Title/Abstract]))) OR (("Antibiotic Prophylaxis"[Mesh]) OR ((((Antibiotic Prophylaxis[Title/Abstract]) OR (Antibiotic Prophylaxi[Title/Abstract])) OR (Antibiotic Premedication[Title/Abstract])) OR (Antibiotic Premedications[Title/Abstract])))) AND (("Invasive Fungal Infections"[Mesh]) OR (((((Invasive Fungal Infection[Title/Abstract]) OR (Invasive Fungal Infections[Title/Abstract])) OR (Invasive Fungal Infection[Title/Abstract])) OR (Invasive Mycoses[Title/Abstract])) OR (Invasive Mycose[Title/Abstract])))) AND ((("Leukemia"[Mesh]) OR ((((((Leukemia[Title/Abstract]) OR (Leukemias[Title/Abstract])) OR (Leucocythaemia[Title/Abstract])) OR (Leucocythaemias[Title/Abstract])) OR (Leucocythemia[Title/Abstract])) OR (Leucocythemias[Title/Abstract]))) OR (("Bone Marrow Transplantation"[Mesh]) OR (((bone marrow transplantation[Title/Abstract]) OR (Bone Marrow Grafting[Title/Abstract])) OR (Bone Marrow Cell Transplantation[Title/Abstract])))) |
| 17 | (("Leukemia"[Mesh]) OR ((((((Leukemia[Title/Abstract]) OR (Leukemias[Title/Abstract])) OR (Leucocythaemia[Title/Abstract])) OR (Leucocythaemias[Title/Abstract])) OR (Leucocythemia[Title/Abstract])) OR (Leucocythemias[Title/Abstract]))) OR (("Bone Marrow Transplantation"[Mesh]) OR (((bone marrow transplantation[Title/Abstract]) OR (Bone Marrow Grafting[Title/Abstract])) OR (Bone Marrow Cell Transplantation[Title/Abstract]))) |
| 16 | ("Bone Marrow Transplantation"[Mesh]) OR (((bone marrow transplantation[Title/Abstract]) OR (Bone Marrow Grafting[Title/Abstract])) OR (Bone Marrow Cell Transplantation[Title/Abstract])) |
| 15 | ((bone marrow transplantation[Title/Abstract]) OR (Bone Marrow Grafting[Title/Abstract])) OR (Bone Marrow Cell Transplantation[Title/Abstract]) |
| 14 | "Bone Marrow Transplantation"[Mesh] |
| 13 | ("Leukemia"[Mesh]) OR ((((((Leukemia[Title/Abstract]) OR (Leukemias[Title/Abstract])) OR (Leucocythaemia[Title/Abstract])) OR (Leucocythaemias[Title/Abstract])) OR (Leucocythemia[Title/Abstract])) OR (Leucocythemias[Title/Abstract])) |
| 12 | (((((Leukemia[Title/Abstract]) OR (Leukemias[Title/Abstract])) OR (Leucocythaemia[Title/Abstract])) OR (Leucocythaemias[Title/Abstract])) OR (Leucocythemia[Title/Abstract])) OR (Leucocythemias[Title/Abstract]) |
| 11 | "Leukemia"[Mesh] |
| 10 | ("Invasive Fungal Infections"[Mesh]) OR (((((Invasive Fungal Infection[Title/Abstract]) OR (Invasive Fungal Infections[Title/Abstract])) OR (Invasive Fungal Infection[Title/Abstract])) OR (Invasive Mycoses[Title/Abstract])) OR (Invasive Mycose[Title/Abstract])) |
| 9 | ((((Invasive Fungal Infection[Title/Abstract]) OR (Invasive Fungal Infections[Title/Abstract])) OR (Invasive Fungal Infection[Title/Abstract])) OR (Invasive Mycoses[Title/Abstract])) OR (Invasive Mycose[Title/Abstract]) |
| 8 | "Invasive Fungal Infections"[Mesh] |
| 7 | (("Pre-Exposure Prophylaxis"[Mesh]) OR ((((Pre Exposure Prophylaxis[Title/Abstract]) OR (Pre-Exposure Prophylaxi[Title/Abstract])) OR (Pre-Exposure Prophylaxis[Title/Abstract])) OR (Pre Exposure Prophylaxis[Title/Abstract]))) OR (("Antibiotic Prophylaxis"[Mesh]) OR ((((Antibiotic Prophylaxis[Title/Abstract]) OR (Antibiotic Prophylaxi[Title/Abstract])) OR (Antibiotic Premedication[Title/Abstract])) OR (Antibiotic Premedications[Title/Abstract]))) |
| 6 | ("Antibiotic Prophylaxis"[Mesh]) OR ((((Antibiotic Prophylaxis[Title/Abstract]) OR (Antibiotic Prophylaxi[Title/Abstract])) OR (Antibiotic Premedication[Title/Abstract])) OR (Antibiotic Premedications[Title/Abstract])) |
| 5 | (((Antibiotic Prophylaxis[Title/Abstract]) OR (Antibiotic Prophylaxi[Title/Abstract])) OR (Antibiotic Premedication[Title/Abstract])) OR (Antibiotic Premedications[Title/Abstract]) |
| 4 | "Antibiotic Prophylaxis"[Mesh] |
| 3 | ("Pre-Exposure Prophylaxis"[Mesh]) OR ((((Pre Exposure Prophylaxis[Title/Abstract]) OR (Pre-Exposure Prophylaxi[Title/Abstract])) OR (Pre-Exposure Prophylaxis[Title/Abstract])) OR (Pre Exposure Prophylaxis[Title/Abstract])) |
| 2 | (((Pre Exposure Prophylaxis[Title/Abstract]) OR (Pre-Exposure Prophylaxi[Title/Abstract])) OR (Pre-Exposure Prophylaxis[Title/Abstract])) OR (Pre Exposure Prophylaxis[Title/Abstract]) |
| 1 | "Pre-Exposure Prophylaxis"[Mesh] |

1. **Cochrane search strategy**

| **ID** | **Search** |
| --- | --- |
| #1 | (Pre Exposure Prophylaxis):ti,ab,kw OR (Pre-Exposure Prophylaxi):ti,ab,kw OR (Pre-Exposure Prophylaxis):ti,ab,kw OR (Pre Exposure Prophylaxis):ti,ab,kw |
| #2 | MeSH descriptor: [Pre-Exposure Prophylaxis] explode all trees MeSH |
| #3 | #1 OR #2 |
| #4 | (Antibiotic Prophylaxis):ti,ab,kw OR (Antibiotic Prophylaxi):ti,ab,kw OR (Antibiotic Premedication):ti,ab,kw OR (Antibiotic Premedications):ti,ab,kw |
| #5 | MeSH descriptor: [Pre-Exposure Prophylaxis] explode all trees MeSH |
| #6 | #4 OR #5 |
| #7 | #3 OR #6 |
| #8 | (Invasive Fungal Infection):ti,ab,kw OR (Invasive Fungal Infections):ti,ab,kw OR (Invasive Fungal Infection):ti,ab,kw OR (Invasive Mycoses):ti,ab,kw OR (Invasive Mycose):ti,ab,kw |
| #9 | MeSH descriptor: [Invasive Fungal Infections] explode all trees MeSH |
| #10 | #8 OR #9 |
| #11 | (Leukemia):ti,ab,kw OR (Leucocythemias):ti,ab,kw OR (Leucocythaemia):ti,ab,kw OR (Leucocythaemias):ti,ab,kw OR (Leucocythemia):ti,ab,kw |
| #12 | MeSH descriptor: [Leukemia] explode all trees MeSH |
| #13 | #11 OR #12 |
| #14 | (bone marrow transplantation):ti,ab,kw OR (Bone Marrow Grafting):ti,ab,kw OR (Bone Marrow Cell Transplantation):ti,ab,kw |
| #15 | MeSH descriptor: [Bone Marrow Transplantation] explode all trees MeSH |
| #16 | #14 OR #15 |
| #17 | #13 OR #16 |
| #18 | #7 AND #10 AND #17 |

1. **Embase search strategy**

| No. | Query |
| --- | --- |
| #18 | #7 AND #10 AND #17 |
| #17 | #13 OR #16 |
| #16 | #14 OR #15 |
| #15 | 'bone marrow transplantation'/exp OR 'allogenic bone marrow transplantation'/exp OR 'autologous bone marrow transplantation'/exp |
| #14 | 'bone marrow transplantation':ti,ab,kw OR 'bone marrow grafting':ti,ab,kw OR 'bone marrow cell transplantation':ti,ab,kw |
| #13 | #11 OR #12 |
| #12 | 'leukemia'/exp |
| #11 | leukemia:ti,ab,kw OR leukemias:ti,ab,kw OR leucocythaemia:ti,ab,kw OR leucocythaemias:ti,ab,kw OR leucocythemia:ti,ab,kw OR leucocythemias:ti,ab,kw |
| #10 | #8 OR #9 |
| #9 | 'systemic mycosis'/exp |
| #8 | 'invasive fungal infections':ti,ab,kw OR 'invasive fungal infection':ti,ab,kw OR 'invasive mycoses':ti,ab,kw OR 'invasive mycose':ti,ab,kw |
| #7 | #3 OR #6 |
| #6 | #4 OR #5 |
| #5 | 'antibiotic prophylaxis'/exp |
| #4 | 'antibiotic prophylaxis':ti,ab,kw OR 'antibiotic prophylaxi':ti,ab,kw OR 'antibiotic premedication':ti,ab,kw OR 'antibiotic premedications':ti,ab,kw |
| #3 | #1 OR #2 |
| #2 | 'pre-exposure prophylaxis'/exp |
| #1 | 'pre-exposure prophylaxi':ti,ab,kw OR 'pre-exposure prophylaxis':ti,ab,kw OR 'pre exposure prophylaxis':ti,ab,kw |
