# Supplementary material for: Network meta-analysis of triazole, polyene, and echinocandin antifungal agents in invasive fungal infection prophylaxis in patients with hematological malignancies
Source: BMC Cancer. 2021 Apr 14;21:404. doi: 10.1186/s12885-021-07973-8 (PMC8048157; doi:10.1186/s12885-021-07973-8)
Supplement: Supplementary file 3 — Additional file 3: Fig. S1. Forest plot of possible and proven IFI. Fig. S2. Forest plot of IA-related possible and proven IFI. Fig. S3. Forest plot of IC-related possible and proven IFI. Fig. S4. Forest plot of all cause mortality. Fig. S5. Forest plot of IFI-related mortality. Fig. S6. Forest plot of AE. Fig. S7. Forest plot of withdrawal due AE. Fig. S8. Forest plot of empirical treatment. Fig. S9. Forest plot of successful treatment. Fig. S10. SUCRA of all drugs for proven and probable IFI [file 12885_2021_7973_MOESM3_ESM.docx]

**
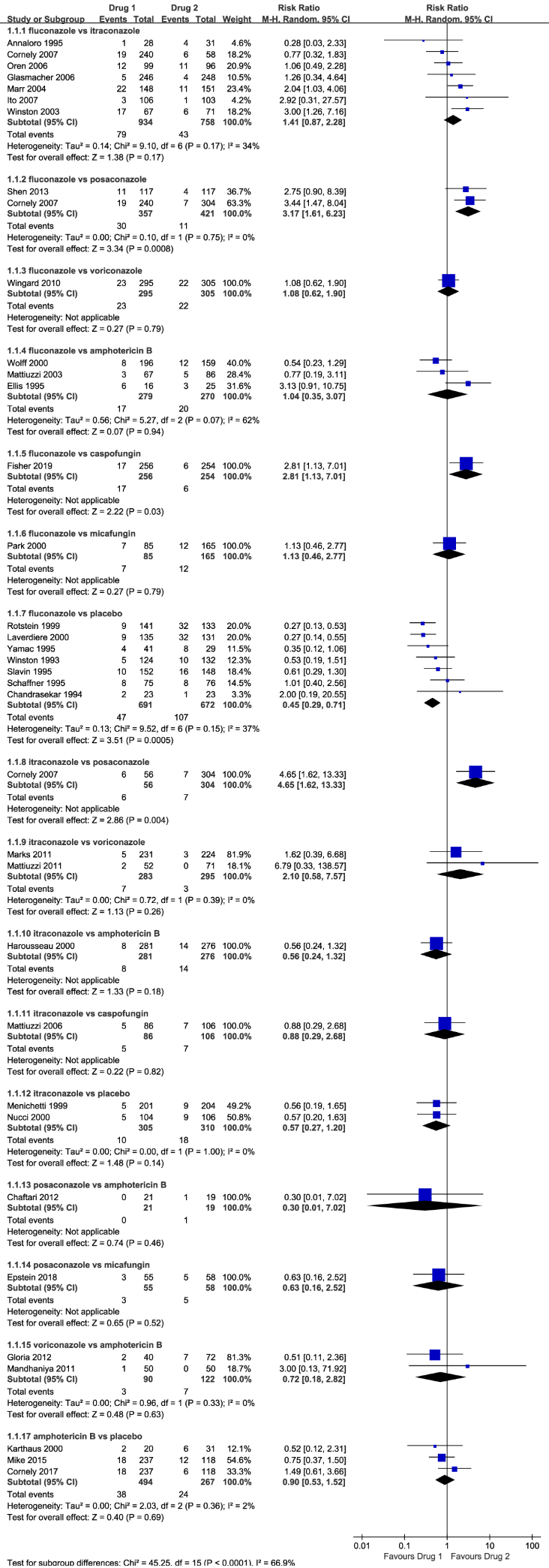
**

**Figure S1. Forest plot of possible and proven IFI**

**
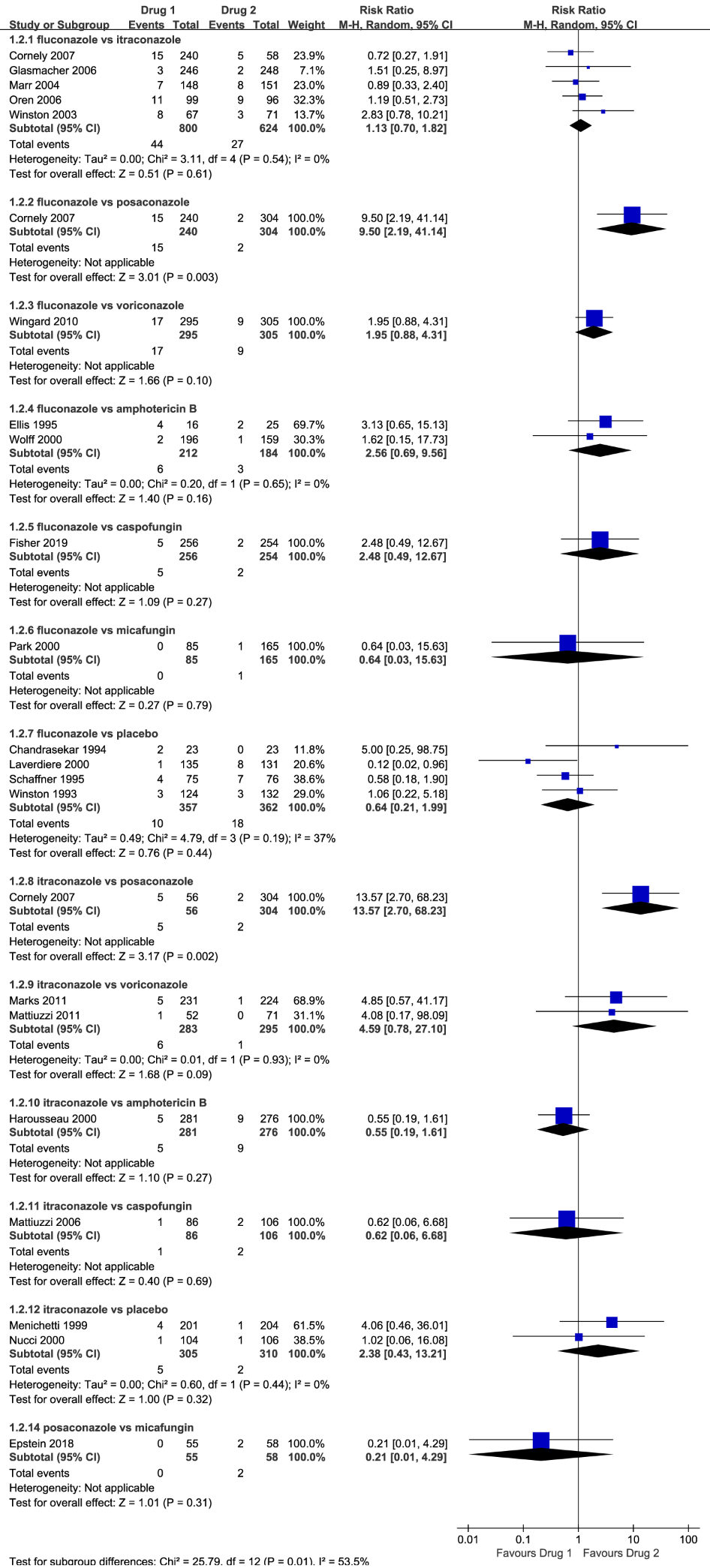
**

**Figure S2. Forest plot of IA-related possible and proven IFI**

**
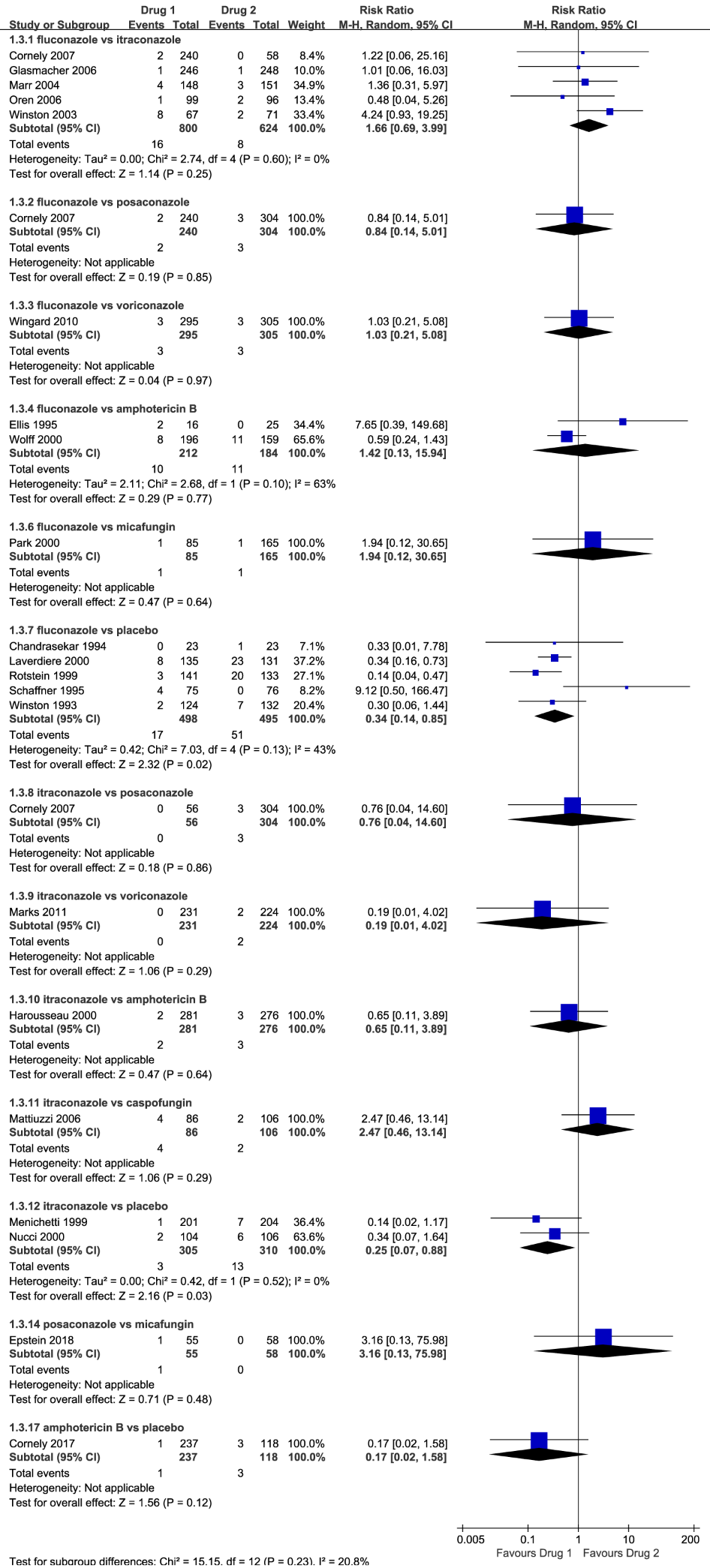
**

**Figure S3. Forest plot of IC-related possible and proven IFI**

**
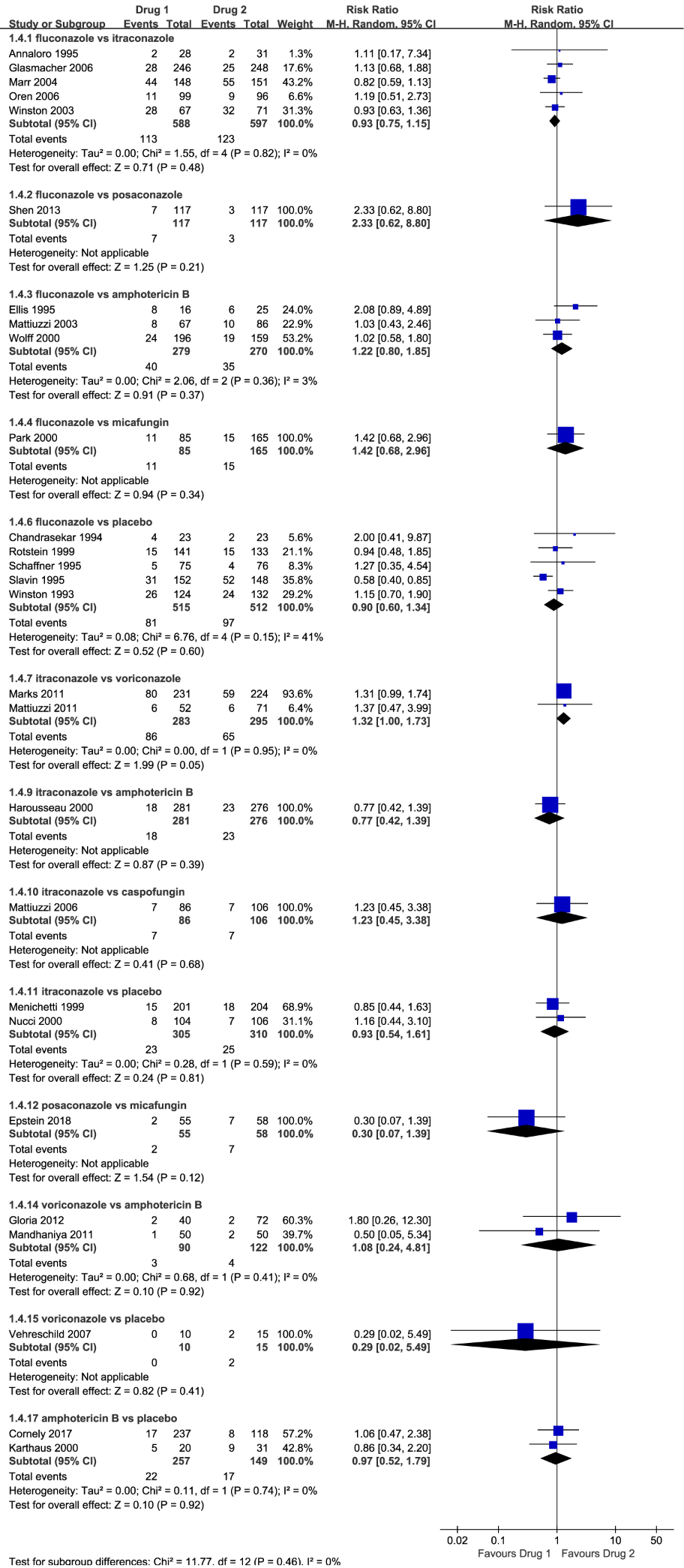
**

**Figure S4. Forest plot of all cause mortality**

**
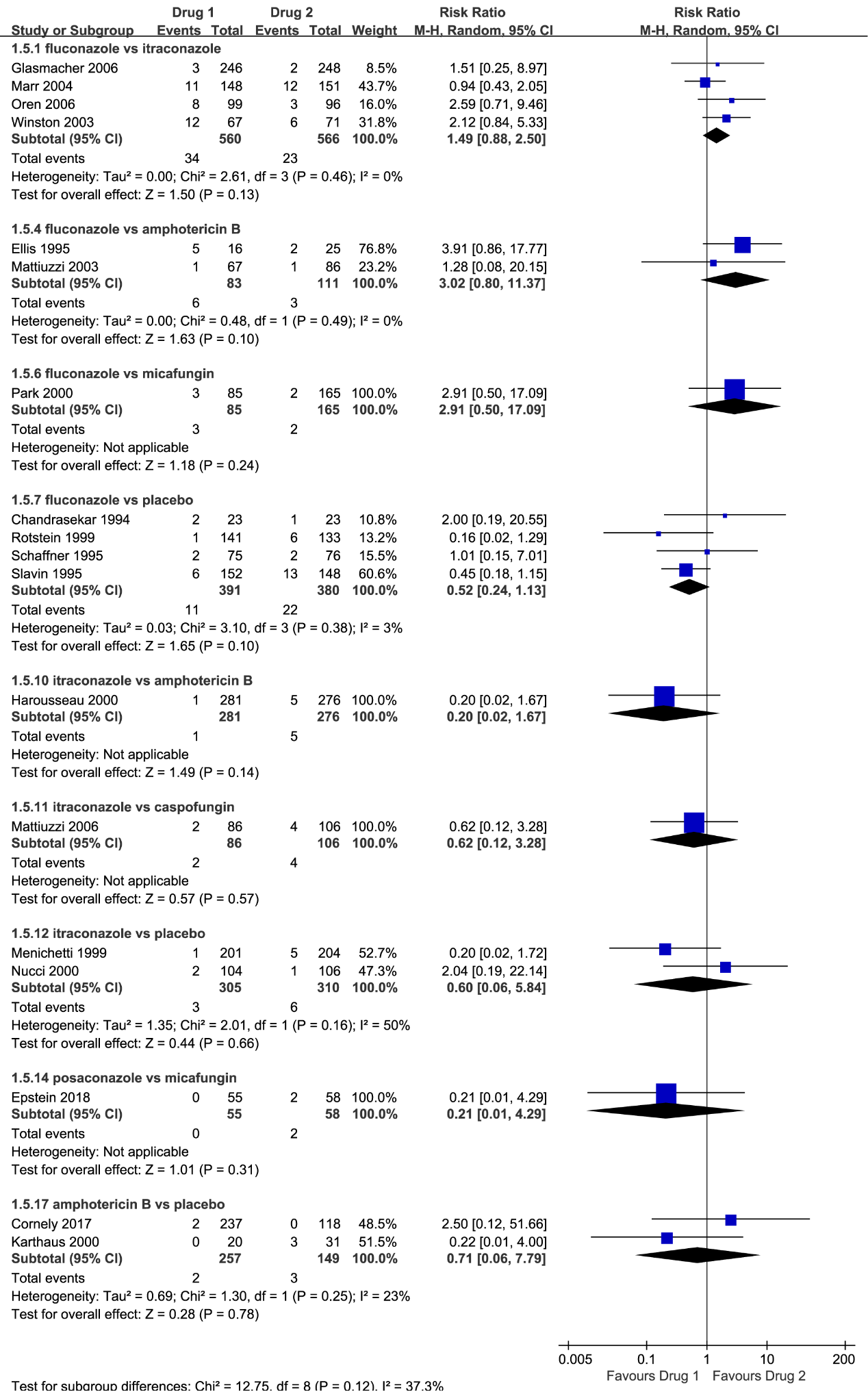
**

**Figure S5. Forest plot of IFI-related mortality**

**
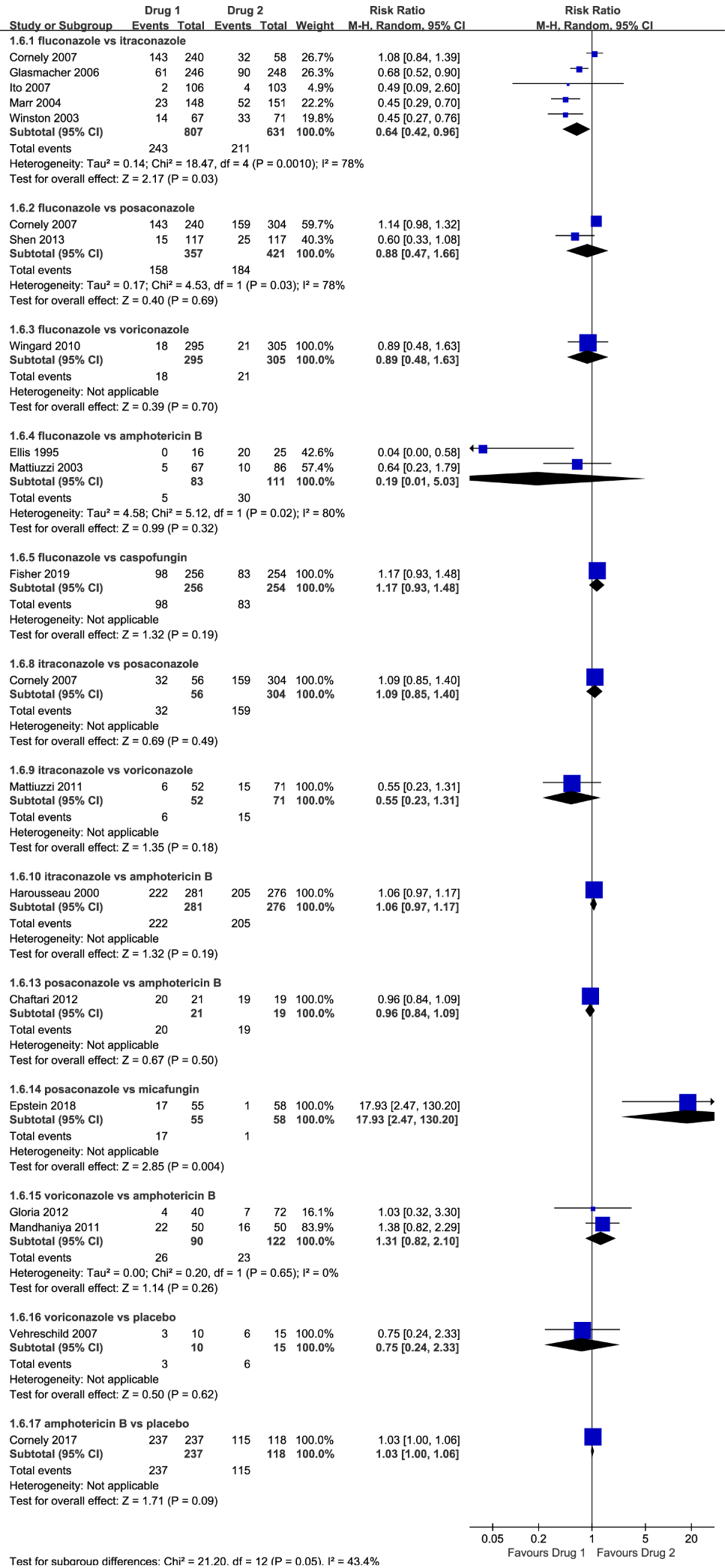
**

**Figure S6. Forest plot of AE**

**
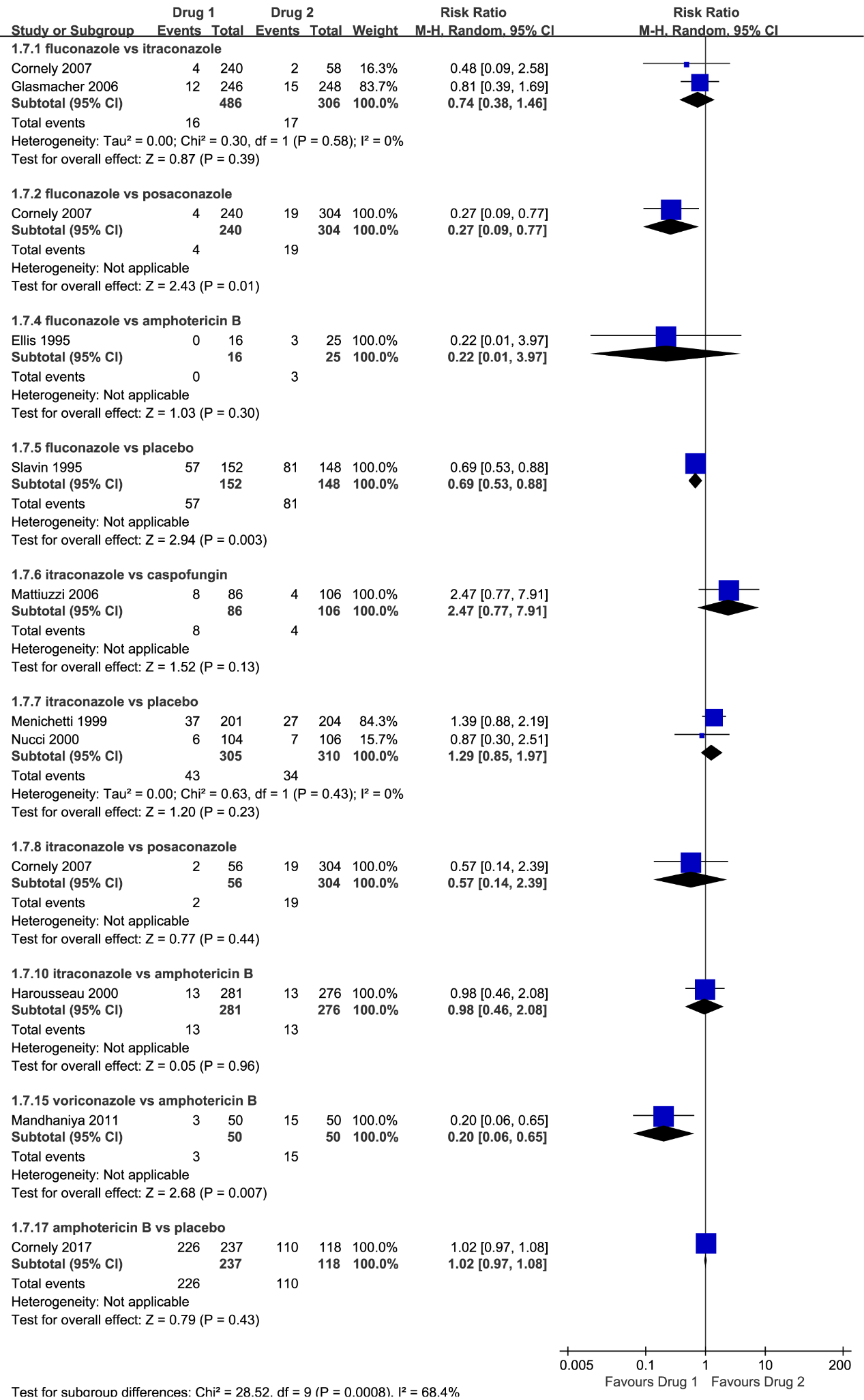
**

**Figure S7. Forest plot of withdrawal due AE**

**
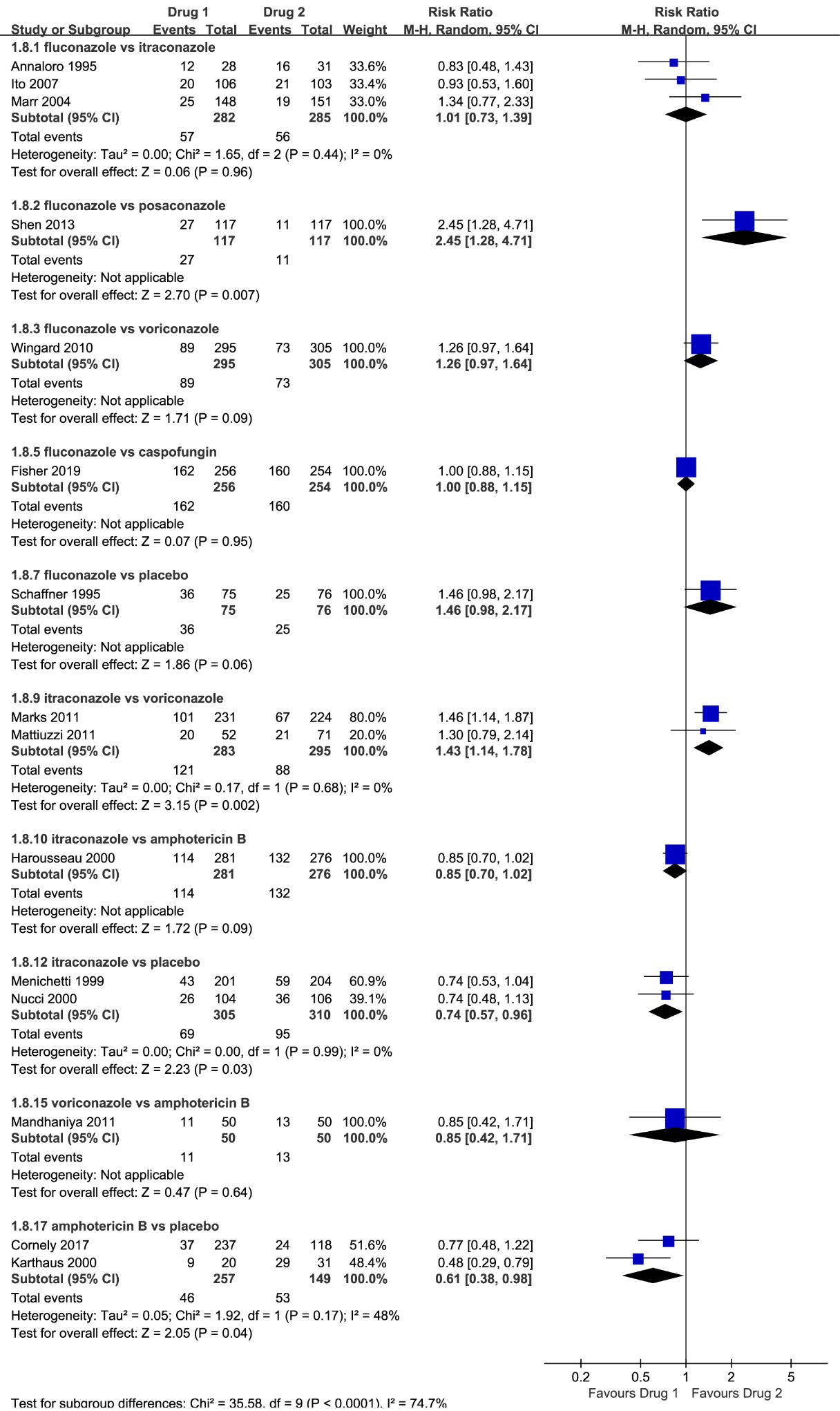
**

**Figure S8. Forest plot of empirical treatment**

**
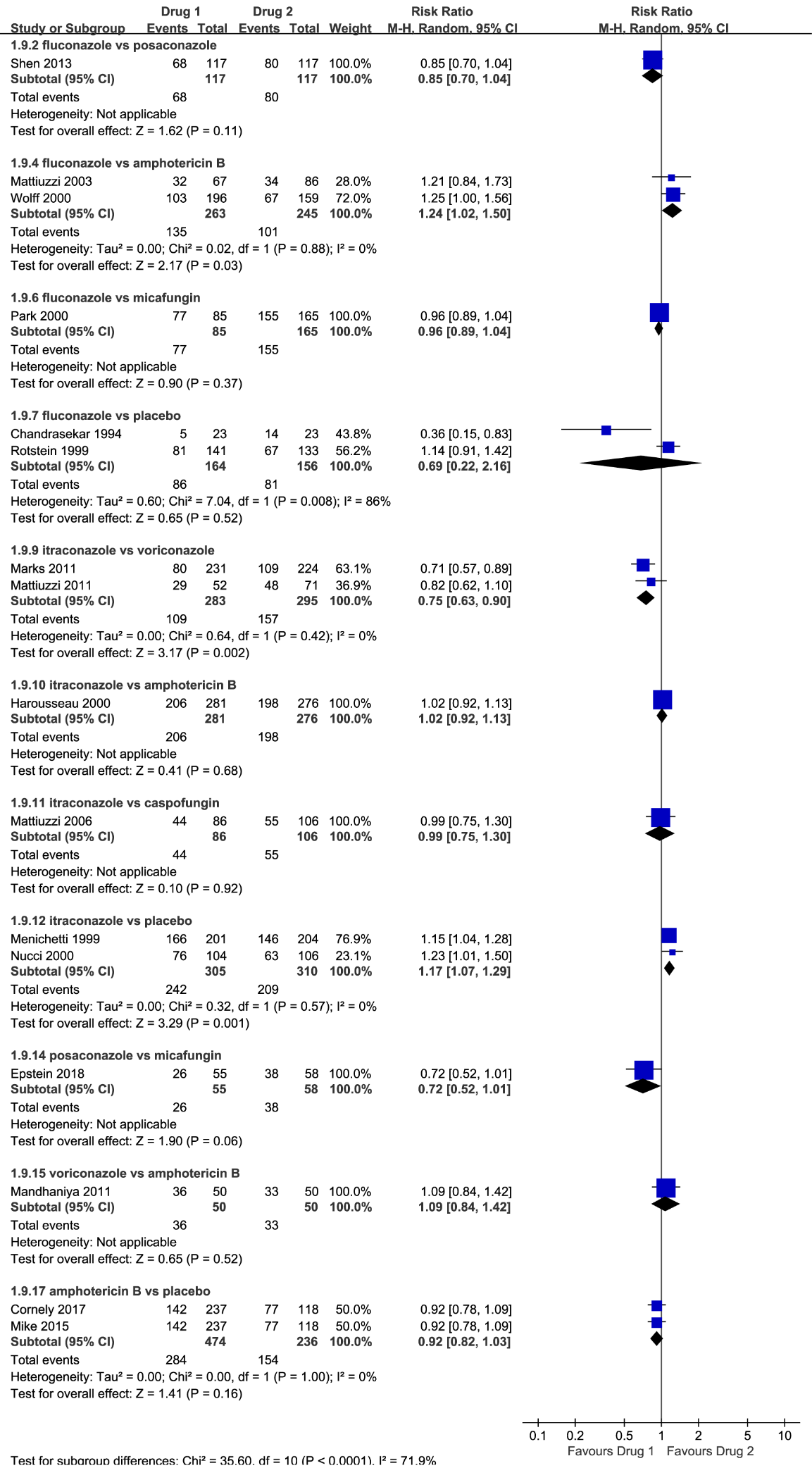
**

**Figure S9. Forest plot of successful treatment**

**
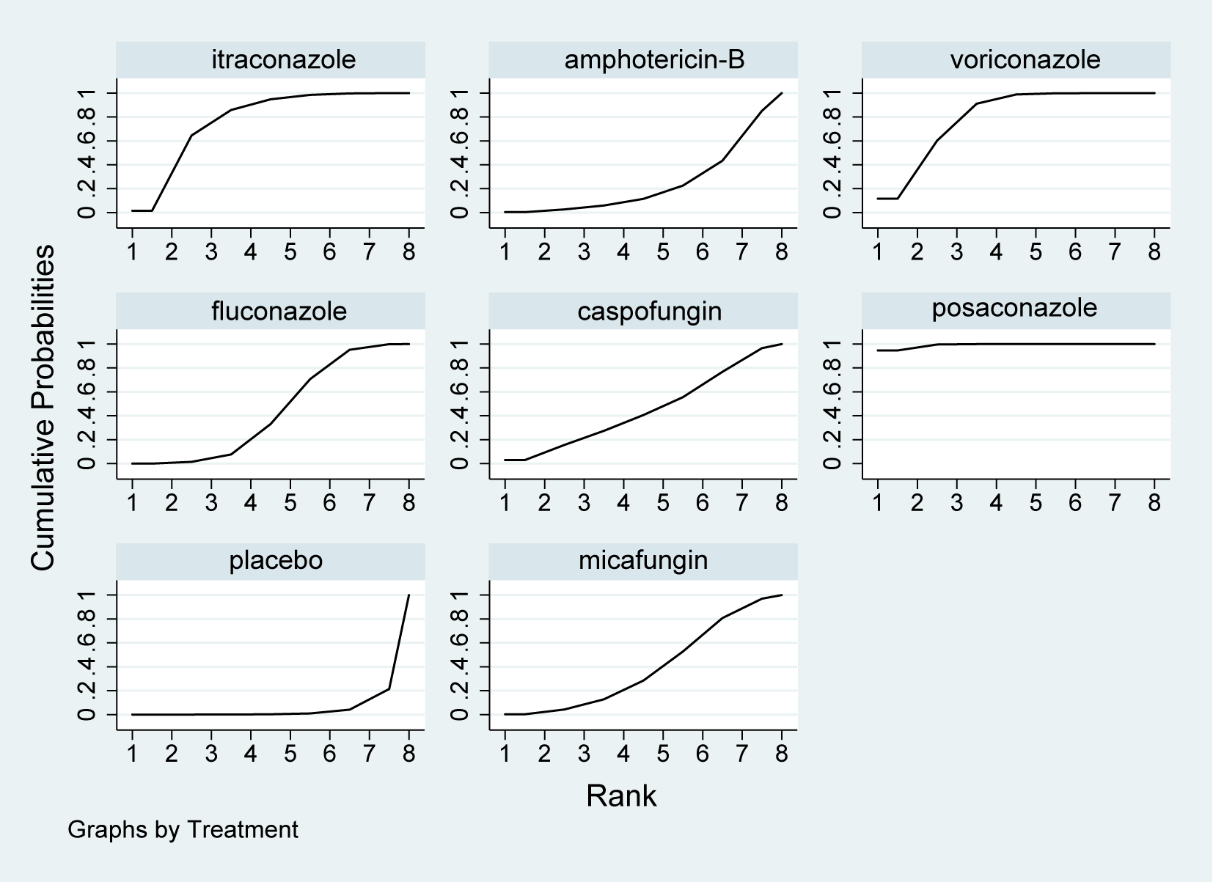
**

**Figure S10. SUCRA of all drugs for proven and probable IFI**
